# Association of Proton Pump Inhibitor Use With Risk of Acquiring Drug-Resistant Enterobacterales

**DOI:** 10.1001/jamanetworkopen.2023.0470

**Published:** 2023-02-23

**Authors:** Roel P. J. Willems, Martijn C. Schut, Anna M. Kaiser, Thomas H. Groot, Ameen Abu-Hanna, Jos W. R. Twisk, Karin van Dijk, Christina M. J. E. Vandenbroucke-Grauls

**Affiliations:** 1 Department of Medical Microbiology and Infection Prevention, Amsterdam Infection and Immunity Institute, Amsterdam University Medical Centers, Academic Medical Center, Amsterdam, the Netherlands; 2 Department of Medical Informatics, Amsterdam Public Health, Amsterdam University Medical Centers, Academic Medical Center, Amsterdam, the Netherlands; 3 Department of Medical Microbiology and Infection Prevention, Amsterdam University Medical Centers, Vrije University Amsterdam, Amsterdam, the Netherlands; 4 Department of Clinical Epidemiology and Data Science, Amsterdam Public Health, Amsterdam University Medical Centers, Amsterdam, the Netherlands; 5 Department of Clinical Medicine and Department of Clinical Epidemiology, Aarhus University, Aarhus, Denmark

## Abstract

**Importance:**

Proton-pump inhibitors (PPIs) have been associated with the risk of colonization with drug-resistant bacteria; however, possible confounding by lifestyle-associated factors and disease severity casts doubt on this association, and whether the risk is dose dependent is not known.

**Objectives:**

To assess the association between PPI use and the risk of acquiring drug-resistant Enterobacterales and to examine interactions with possible microbiome-altering agents.

**Design, Setting, and Participants:**

This nested case-control study involved 2239 hospitalized adult (aged ≥18 years) patients identified from the microbiology laboratory database of Amsterdam University Medical Centers between December 31, 2018, and January 6, 2021. Patients in the case group had newly detected extended-spectrum β-lactamase (ESBL)– or carbapenemase-producing Enterobacterales (identified by clinical specimens). Risk-set sampling was used to assign patients with negative results for ESBL- and carbapenemase-producing Enterobacterales to the control group, who were then matched on a 5:1 ratio with patients in the case group by age and culture date. A second validation case-control study included matched pairs (1:1 ratio; 94 in each group) of patients who were prospectively enrolled.

**Exposures:**

Proton pump inhibitor use and clinical data at 30 days (primary exposure) and 90 days (secondary exposure) before the date of culture.

**Main Outcomes and Measures:**

Adjusted incidence rate ratios (aIRRs) of ESBL- or carbapenemase-producing Enterobacterales acquisition by PPI dose and time risk windows (30 days for the primary outcome and 90 days for the secondary outcome) were estimated using conditional logistic regression models.

**Results:**

Among 2239 hospitalized patients (51.1% male; mean [SD] age, 60.9 [16.7] years), 374 were in the case group (51.6% male; mean [SD] age, 61.1 [16.5] years) and 1865 were in the matched control group (51.0% male; mean [SD] age, 60.9 [16.7] years). The aIRR for PPI use overall was 1.48 (95% CI, 1.15-1.91) at 30 days. Sensitivity analyses and the analysis of the pair-matched study with prospectively enrolled patients (aIRR, 2.96, 95% CI, 1.14-7.74) yielded similar results; findings were consistent in subgroups and corroborated by a negative-control exposure analysis. No association with microbiome-disturbing agents was found; laxatives and antibiotics were independently associated with a more than 2-fold increase in the risk of acquisition (antibiotics: aIRR, 2.78 [95% CI, 2.14-3.59]; laxatives: aIRR, 2.26 [95% CI. 1.73-2.94]).

**Conclusions and Relevance:**

In this study, after careful control for confounding and sensitivity analyses, PPI use was associated with increases in the risk of acquiring ESBL- or carbapenemase-producing Enterobacterales among adult hospitalized patients. These findings emphasize the need for judicious use of PPIs.

## Key Points

**Question:**

Are proton pump inhibitors (PPIs) associated with increased risk of acquiring extended-spectrum β-lactamase (ESBL)– or carbapenemase-producing Enterobacterales in hospitalized patients?

**Findings:**

In this case-control study of 2239 adult hospitalized patients with and without ESBL- or carbapenemase-producing Enterobacterales, after controlling for possible confounding factors, patients who received PPIs within the previous 30 days had a 1.48-fold increased risk of acquiring ESBL- or carbapenemase-producing Enterobacterales compared with those who did not receive PPIs.

**Meaning:**

These findings reinforce the need to promote judicious use of PPIs to mitigate the risk of acquiring drug-resistant Enterobacterales among hospitalized patients.

## Teaser

This case-control study assesses the association between the use of proton pump inhibitors and the risk of acquiring drug-resistant Enterobacterales and examines interactions with possible microbiome-altering agents among adult hospitalized patients.

## Introduction

Resistance to extended-spectrum β-lactamases (ESBLs) and carbapenemases is recognized as an increasingly major problem for public health.^1,2,3^ Concerns have been raised that proton pump inhibitors (PPIs), which are widely overused (approximately 30%-50% are overprescribed^4,5^), may increase the risk of colonization with drug-resistant microorganisms.

A recent systematic review and meta-analysis^6^ summarized available evidence and found 70% higher odds of multidrug-resistant bacterial colonization with the use of PPIs. However, several studies included in this meta-analysis^6^ did not primarily examine the consequences of PPI use as part of the exposure-outcome association, which led to varying control for confounding and modifying factors. Confounding owing to disease severity and unhealthy lifestyle factors may augment the risk seen in previous studies because PPI treatment is common among older patients with multimorbidity.^7,8^ To our knowledge, the implications of dose or duration of PPI treatment have not yet been studied. Mechanistically, reduction of gastric acidity may lead to increased gastric passage of pathogens or viable exogenous drug-resistant strains, delayed gastric emptying, increased bacterial translocation, and dysbiosis, resulting in intestinal colonization or infection.^9,10,11,12,13^ To address these issues, we conducted a nested case-control study to investigate the association between the use of PPIs and the risk of acquiring ESBL- or carbapenemase-producing Enterobacterales in patients hospitalized in 2 tertiary care centers in Amsterdam, the Netherlands. We aimed to verify the association of PPI use with acquisition of drug-resistant Enterobacterales, assess a possible dose-response association, and examine potential interactions with other microbiome-altering medications.

## Methods

### Setting, Design, and Data Sources

The Vrije Universiteit Medical Center Research Ethics Committee approved this study and granted a waiver of informed consent for the retrospectively conducted case-control study because we used existing data that were deidentified. For the prospectively conducted case-control study, we obtained written informed consent from all participants. This study followed the Strengthening the Reporting of Observational Studies in Epidemiology (STROBE)^14^ guideline and the Reporting of Studies Conducted Using Observational Routinely Collected Health Data Statement for Pharmacoepidemiology (RECORD-PE)^15^ guideline for case-control studies. We used the nested case-control approach as an alternative design to randomization, and we matched by design to increase efficiency.^16^

The study included a cohort of 2239 adult (aged ≥18 years) patients admitted to the 1800-bed Amsterdam University Medical Centers, which comprise 2 tertiary care centers under common management (eFigure 1 in Supplement 1). Patients with and without ESBL- or carbapenemase-producing Enterobacterales were identified from the Clinical Microbiology Laboratory Information System between December 31, 2018, and January 6, 2021. This system contains data on all specimens submitted to the microbiology laboratory, including patient identification data, specimen type, culture results, antibiotic susceptibility data, and molecular analysis results. We included patients with 1 or more specimens collected for culture at admission or during admission to any hospital department (including the emergency department). According to hospital policies, patients at high risk of colonization are screened for carriage of ESBL- or carbapenemase-producing Enterobacterales by fecal swabs (eMethods 1 in Supplement 1). No outbreaks occurred during the study period. Both centers apply standard contact isolation precautions for patients with ESBL- and/or carbapenemase-producing Enterobacterales colonization and/or infection.

### Case Identification

Patients in the case group had positive results for ESBL- or carbapenemase-producing Enterobacterales that was newly detected in selected specimens submitted to the microbiology laboratory during hospital admission. Standard laboratory culture methods and techniques based on established guidelines were used to identify patients with ESBL- or carbapenemase-producing Enterobacterales (eMethods 1 in Supplement 1).^17^ We excluded patients with a known history of previous acquisition of ESBL- or carbapenemase-producing Enterobacterales, and only patients with no ESBL- or carbapenemase-producing Enterobacterales detected in cultures between January 1, 2015, and December 31, 2017, were included. Culture specimens eligible for inclusion were feces, sputum, urine, blood, and ascites yielding ESBL- or carbapenemase-producing Enterobacterales. Patients could have positive results at more than 1 culture site. We did not differentiate carriage from infection, given that patients with infection are also carriers.^18^

### Control Population

For each patient in the case group, we randomly assigned up to 5 patients without ESBL- or carbapenemase-producing Enterobacterales (control group) who were identified from the laboratory system via risk-set sampling without replacement.^19^ Individuals in the control group were inpatients from whom specimens were sent for culture on or close to the same day as those in the case group, provided that these cultures did not yield ESBL- and/or carbapenemase-producing Enterobacterales.^20^ Using a greedy matching algorithm, patients in the control group were matched by index date (date the patient had a positive test result; 15-day caliper) and age (5-year caliper) to increase comparability between the case and control groups with respect to correlates of age. Patients in the control group who were at risk over time could reenter the cohort at a later time and be assigned to the case group.

### Clinical Data Extraction

We used clinical data from the Amsterdam University Medical Centers Research Data Platform. This data warehouse contains integrated data from several sources, including electronic patient information (eg, demographic characteristics, medical diagnoses, and prescriptions), and is established prospectively during standard care (eMethods 1 in Supplement 1). An independent data manager extracted the study data.

### Exposure Assessment

The primary exposure was PPI use within 30 days before the index date, with continuous PPI treatment according to prescribed drug dispensing data and identified by Anatomical Therapeutic Chemical (ATC) Classification System^21^ code A02BC in the database’s pharmacy records (which include both inpatient and outpatient prescription use) (eTable 1B in Supplement 1). The secondary exposure was PPI use within 90 days before the index date. We categorized daily frequency of PPI use as once daily or twice daily dosing within the time risk window; most clinical doses of PPIs were similar to the defined daily dose. Because insufficient numbers of patients used histamine 2 receptor antagonists, we assessed risk but not dose response for these gastric acid suppressants.

### Covariates

To account for confounding factors, we selected covariates based on the literature and integrated these covariates in a directed acyclic graph (eMethods 2 and eFigure 2 in Supplement 1).^22^ We extracted data on the following variables: age at index admission, sex, body mass index (BMI; calculated as weight in kilograms divided by height in meters squared; measured proximal to index date), hospital department, admission from another health care facility, time from admission until index date, data on admission to and length of stay in the intensive care unit (ICU), and receipt of a surgical procedure and/or solid organ or stem cell transplant within the past 90 days. As a proxy for disease severity, we used the number of hospitalization days during the previous 6 months, scores on the Katz Index of Independence in Activities of Daily Living (range, 0-6, with higher scores indicating greater independence),^23^ and scores on the Charlson Comorbidity Index (CCI).^24^ Prevalent diagnoses were identified using *International Statistical Classification of Diseases and Related Health Problems, Tenth Revision *codes in the electronic database (eTable 1A in Supplement 1).

From pharmacy records (which included preadmission medication use), we extracted data on antibiotic use, both before hospital admission and during hospitalization. In addition, we extracted data on the use of nonantibiotic agents that are known to disturb the intestinal microbiome (metformin, laxative, and immunosuppressant medications).^25^ For antibiotic use, we calculated the number of days of in-hospital antibiotic exposure. The use of antirheumatic agents and a diagnosis of inflammatory bowel disease were included as surrogate markers of immunosuppression. Use of medication applied to the time risk window before the index date. No data on patient race and ethnicity were available due to privacy regulations.

### Statistical Analysis

For the primary analysis, we used multiple conditional logistic regression models for matched sets to estimate the incidence rate ratios (IRRs) and 95% CIs of ESBL- and carbapenemase-producing Enterobacterales acquisition by PPI dose.^26,27^ Confounding was handled in 2 steps. First, adjustment was made a priori for confounding factors,^22,28^ including sex, BMI, presence of inflammatory bowel disease, CCI score, and length of ICU stay. Second, to verify the results, we also adjusted for (1) previous hospitalization or receipt of a surgical procedure or transplant as a proxy for unmeasured confounding by underlying disease and (2) use of cephalosporin in lieu of previous hospitalization or receipt of a surgical procedure or transplant.^29,30^

To account for a higher baseline colonization risk, we performed stratified analyses (predefined stratification: sex and CCI score [≤2 vs >2]; post hoc stratification: cancer diagnosis and a proxy for frailty [Katz index]) and tested for interactions with PPI treatment. In addition, interaction was analyzed for agents that potentially disturb the microbiome (antibiotics, laxatives, and metformin) by calculating the relative excess risk due to interaction (eMethods 1 in Supplement 1).^14^ Similarly, the risk of acquiring ESBL- or carbapenemase-producing Enterobacterales with use of these other agents was explored.

To assess whether results were robust, we performed preplanned sensitivity analyses by (1) evaluating the consequences of a set of residual confounders and corroborating the primary analysis in a second 1:1 matching-pairs case-control study of prospectively enrolled patients (94 in each group), (2) excluding patients admitted from another health care facility because these patients may have been previously colonized, (3) calculating the risk of acquisition for thiazide diuretic and bisphosphonate agents (these drugs were used as negative control exposure[s] because, to our knowledge, they have never been associated with increases in the risk of acquiring enteric pathogens or drug-resistant strains^31^), (4) accounting for case mix by restricting the analysis to patients with ESBL- or carbapenemase-producing Enterobacterales cultured from a normally sterile culture site (blood, ascites, and/or other sterile fluids; these positive cultures were considered a proxy for infection) and their matched patients in the control group, and (5) accounting for case mix by restricting the analysis to patients in the case group with fecal carriage only.

The 1:1 matching-pairs design and analysis paralleled those of the main study (eMethods 1 in Supplement 1). To assess potential bias from factors not recorded in the hospital database, patients included in this analysis were asked to complete a short questionnaire on lifestyle-related factors and travel in the past 6 months.

Because not all patients in the control group had undergone fecal screening for carriage, we assessed the consequences of misclassification of patients in the control group using quantitative bias analysis.^32,33^

We used multiple imputation by chained equations to account for covariates with 5% missing data or more (25.6% of patients were missing BMI information) (eMethods 1 in Supplement 1).^34^ Analyses with imputed data were compared with complete-case analysis.

Data were managed using R software, version 4.0.3 (R Foundation for Statistical Computing), and analyzed using Stata SE, version 15.0 (StataCorp LLC). The case-control sets were generated using IronPython scripting in the Spotfire analytics platform (TIBCO Software Inc). Statistical precision was determined with 95% CIs. Precision was explored a priori based on the estimated sample size (eMethods 1 in Supplement 1).

## Results

### Patient Characteristics

Patient characteristics are presented in Table 1 and eTables 2 and 3 in Supplement 1. Among 2239 patients, 1145 (51.1%) were male and 1094 (48.9%) were female, with a mean [SD] age of 60.9 [16.7] years. A total of 270 women (24.7%) and 314 men (27.4%) used PPIs, with pantoprazole sodium (ATC code A02BC02) accounting for 72.8% of PPI use. The sample comprised 374 patients in the case group (193 male [51.6%] and 181 female [48.4%]; mean [SD] age, 61.1 [16.5] years) and 1865 patients in the control group (952 male [51.0%] and 913 female [49.0%]; mean [SD] age, 60.9 [16.7] years); 98.9% of patients in the case group were each matched to 5 patients in the control group (eTable 4 and eFigure 3 in Supplement 1). A total of 61 patients (16.3%) in the case group were culture-positive within 48 hours of admission. Patients in the case group were more likely to have prolonged hospitalization than those in the control group (median [IQR] previous hospital days, 3 [1-8] vs 9 [3-24]). Classic risk factors for ESBL- and carbapenemase-producing Enterobacterales, such as burden of chronic diseases (mainly kidney and cardiovascular), antibiotic use, and other markers of disease severity, were more prevalent among those in the case group.

**Table 1.** Clinical Characteristics of Patients

Characteristic	Patients, No. (%)	
	Case group (n = 374)^a^	Control group (n = 1865)
Age at index date, y^b^		
Mean (SD)	61.1 (16.5)	60.9 (16.7)
Median (IQR) [range]	64.0 (51.0-74.0) [19.0-93.0]	63.0 (50.0-74.0) [21.0-97.0]
Group		
<45	75 (20.1)	355 (19.0)
45-64	121 (32.4)	638 (34.2)
≥65	178 (47.6)	872 (46.8)
Sex		
Female	181 (48.4)	913 (49.0)
Male	193 (51.6)	952 (51.0)
Hospital department		
Medical	299 (79.9)	1555 (83.4)
Surgical	50 (13.4)	220 (11.8)
Emergency	25 (6.7)	90 (4.8)
Admission from other health care facility^c^	41 (11.0)	145 (7.8)
Culture site (≥1)^d^		
Feces	181 (48.4)	479 (25.7)
Sterile fluid^e^	52 (13.9)	622 (33.4)
Urine or sputum	175 (46.8)	764 (41.0)
BMI		
Mean (SD)	26.7 (6.1)	26.3 (5.3)
Group		
<25	138 (44.8)	591 (43.8)
25-29.9	94 (30.5)	461 (34.1)
≥30	76 (24.7)	298 (22.1)
Missing	66 (17.6)	515 (27.6)
CCI score		
Median (IQR)	2 (1-5)	1 (0-3)
Group		
0	117 (31.3)	847 (45.4)
1-2	101 (27.0)	513 (27.5)
>2	156 (41.7)	505 (27.1)
Coexisting disease^f^		
Myocardial infarction	42 (11.2)	136 (7.3)
Congestive heart failure	62 (16.6)	132 (7.1)
Peripheral vascular disease	37 (9.9)	113 (6.1)
Neurological disease	50 (13.4)	159 (8.5)
Chronic pulmonary disease	34 (9.1)	174 (9.3)
Rheumatological disease	14 (3.7)	67 (3.6)
Peptic ulcer disease	4 (1.1)	12 (0.6)
Liver disease	35 (9.4)	62 (3.3)
Inflammatory bowel disease	26 (7.0)	97 (5.2)
Diabetes	93 (24.9)	242 (13.0)
Kidney disease	97 (25.9)	222 (11.9)
Cancer	133 (35.6)	630 (33.8)
Katz index score, mean (SD)^g^	5.4 (1.2)	5.6 (1.1)
Previous hospitalization (<6 mo)	139 (37.2)	560 (30.0)
Previous hospitalization with cumulative LOS >7 d	77 (20.6)	158 (8.5)
Previous ICU admission	38 (10.2)	65 (3.5)
Days in the ICU, median (IQR)	5 (2-10)	3 (1-5)
Previous surgical procedure (<90 d)	75 (20.1)	194 (10.4)
Previous solid organ or stem cell transplant (<90 d)	29 (7.8)	60 (3.2)
Medication^h^		
Antirheumatic	23 (6.1)	103 (5.5)
Metformin	27 (7.2)	92 (4.9)
Laxative	170 (45.5)	434 (23.3)
Immunomodulator^i^	127 (34.0)	473 (25.4)
Antibiotic (≥1)	186 (49.7)	447 (24.0)
PPI and/or H2RA^j^	132 (35.3)	452 (24.2)
Acid suppression^k^		
None	242 (64.7)	1413 (75.8)
H2RA	4 (1.1)	13 (0.7)
PPI dosing		
Once daily	106 (28.3)	379 (20.3)
Twice daily	22 (5.9)	60 (3.2)

Abbreviations: BMI, body mass index (calculated as weight in kilograms divided by height in meters squared; measured proximal to index date); CCI, Charlson Comorbidity Index; H2RA, histamine 2 receptor antagonist; ICU, intensive care unit; LOS, length of stay; PPI, proton pump inhibitor.

^a^ Of the 374 patients in the case group, 61 (16.3%) had positive results at 48 hours or less after admission, and 313 (83.7%) had positive results at more than 48 hours after admission.

^b^ Matching variable.

^c^ Admitted from a long-term care facility, hospital, or rehabilitation clinic.

^d^ Because patients could have positive results for more than 1 culture site, percentages exceed 100%.

^e^ Blood, ascites, and/or other sterile fluids.

^f^ Diabetes with and without organ complications. Neurological diseases included cerebrovascular disease, dementia, and/or hemiplegia. The incidence of AIDS was less than 1% in both the case and control groups and was therefore not presented.

^g^ Scores on the Katz Index of Independence in Activities of Daily Living range from 0 to 6, with higher scores indicating greater independence.

^h^ Medication use refers to the 30-day period before the index date; categories are not mutually exclusive.

^i^ Oral glucocorticoid, antineoplastic, or antirheumatic drugs (Anatomical Therapeutic Chemical Classification System codes are listed in eTable 1 in Supplement 1).

^j^ Use of PPIs with or without H2RAs. Few patients used other antacids; 4 patients (1.6%) in the case group and 13 patients (0.9%) in the control group used H2RAs.

^k^ Patients who used H2RAs did not use PPIs simultaneously; however, patients who used PPIs could also use H2RAs.

### Primary Analysis

Unadjusted and adjusted IRRs (aIRRs) from the primary analysis are shown in Table 2. The adjusted risk of acquiring ESBL- or carbapenemase-producing Enterobacterales with PPI use (model 1, adjusted for sex, BMI, CCI score, presence of inflammatory bowel disease, and total length of ICU stay) was nearly 50% higher than the risk without PPI use (PPI use overall at 30 days: aIRR, 1.48; 95% CI, 1.15-1.91). At 30 days, the aIRRs were 1.44 (95% CI, 1.10-1.89) for once-daily PPI dosing and 1.75 (95% CI, 1.03-2.97) for twice-daily dosing. The wide 95% CI of the latter estimate was probably due to the small number of patients using the twice-daily dose, which precluded the ability to reach definite conclusions about a possible dose-response association. Adjustment for cephalosporin use (model 2) or previous hospitalization and surgical procedure in the past 90 days (model 3) did not substantially change the aIRRs (model 2: aIRR for PPI use overall at 30 days, 1.43 [95% CI, 1.11-1.85]; model 3: aIRR for PPI use overall at 30 days, 1.38 [95% CI, 1.06-1.80]). Use of histamine 2 receptor antagonists was not associated with an increased risk in these analyses (eg, model 1 at 30 days: aIRR, 1.45; 95% CI, 0.45-4.67).

**Table 2.** Incidence Rate Ratios for the Association Between PPI Use and Acquisition of ESBL- or Carbapenemase-Producing Enterobacterales^a^

Exposure window^b^	Patients, No. (%)		IRR (95% CI)			
	Case group (n = 374)	Control group (n = 1865)	Unadjusted	Model 1^c^	Model 2^d^	Model 3^e^
**0-30 d**						
PPI overall^f^	132 (35.3)	452 (24.2)	1.74 (1.37-2.22)	1.48 (1.15-1.91)	1.43 (1.11-1.85)	1.38 (1.06-1.80)
Acid suppression						
None	242 (64.7)	1413 (75.8)	1 [Reference]	1 [Reference]	1 [Reference]	1 [Reference]
H2RA	4 (1.1)	13 (0.7)	1.79 (0.58-5.52)	1.45 (0.45-4.67)	1.45 (0.44-4.79)	1.19 (0.36-3.89)
PPI dose						
Once daily	106 (28.3)	379 (20.3)	1.67 (1.29-2.17)	1.44 (1.10-1.89)	1.39 (1.06-1.83)	1.35 (1.03-1.78)
Twice daily	22 (5.9)	60 (3.2)	2.17 (1.30-3.62)	1.75 (1.03-2.97)	1.71 (1.01-2.91)	1.64 (0.96-2.80)
**0-90 d**						
PPI overall^g^	149 (39.8)	525 (28.2)	1.74 (1.37-2.21)	1.49 (1.16-1.92)	1.44 (1.12-1.85)	1.32 (1.02-1.71)
Acid suppression						
None	225 (60.2)	1340 (71.8)	1 [Reference]	1 [Reference]	1 [Reference]	1 [Reference]
H2RA	5 (1.3)	16 (0.9)	1.85 (0.68-5.10)	1.56 (0.55-4.43)	1.47 (0.51-4.30)	1.25 (0.43-3.60)
PPI dose						
Once daily	118 (31.6)	437 (23.4)	1.66 (1.28-2.14)	1.44 (1.10-1.88)	1.39 (1.06-1.82)	1.33 (1.01-1.75)
Twice daily	26 (7.0)	73 (3.9)	2.19 (1.36-3.51)	1.80 (1.10-2.95)	1.69 (1.03-2.78)	1.66 (1.02-2.76)

Abbreviations: ESBL, extended-spectrum β-lactamase; H2RA, histamine 2 receptor antagonist; IRR, incidence rate ratio; PPI, proton pump inhibitor.

^a^ Patients in the case and control groups were matched on age and date of the culture.

^b^ Patients in the case and control groups were assigned to mutually exclusive groups based on H2RA and PPI exposure for the dose variable. Data from patients using H2RAs (4 patients [1.1%] in the case group and 13 patients [0.7%] in the control group for the 0-30–day risk window and 5 patients [1.3%] in the case group and 16 patients [0.9%] in the control group for the 0-90–day risk window) were suppressed but included in the regression model for proper estimation of the exposure effects. The reference variable was nonuse of PPIs and/or H2RAs for all comparisons.

^c^ Model 1 was conditioned on matched sets (ie, age) and adjusted for sex (male or female), body mass index (continuous), Charlson Comorbidity Index score (continuous), inflammatory bowel disease (yes or no), and total length of intensive care unit stay (continuous).

^d^ Model 2 was adjusted for all variables in model 1 plus cephalosporin use.

^e^ Model 3 was adjusted for all variables in model 2 plus previous hospitalization and previous surgical procedure (<90 days).

^f^ Use of PPIs with or without H2RAs 30 days before the index date (including a few patients who used H2RAs alone).

^g^ Use of PPIs with or without H2RAs 90 days before the index date (including a few patients who used H2RAs alone).

### Secondary Analyses

Stratification by sex, high CCI score (>2), diagnosis of cancer, and nonindependence in activities of daily living using conventional logistic regression analysis^26^ yielded estimates that were not different overall (eTable 5 in Supplement 1). There were no (multiplicative) interactions with PPI use (eg, CCI score >2 vs ≤2: aIRR, 1.43 [95% CI, 0.99-2.06] vs 1.57 [95% CI, 1.13-2.19]; *P* = .69 for interaction; cancer diagnosis vs no cancer diagnosis: aIRR, 1.79 [95% CI, 1.17-2.76] vs 1.65 [95% CI, 1.23-2.23]; *P* = .75 for interaction; both adjusted for sex). To determine whether the estimates were valid, results from these unconditional models were compared with those from conditional models. Results from the unconditional and conditional models were similar (eTable 5 in Supplement 1).^35^

Adjustment for proxies of disease severity (CCI score, surgical procedures, length of stay in the ICU, and hospitalization days in the past 6 months) attenuated the unadjusted estimate of the IRR for acquisition of ESBL- or carbapenemase-producing Enterobacterales without negating the risk. Further control for markers of disease severity or factors that reflected differences associated with PPI treatment did not substantially change the adjusted estimates.

### Microbiome-Disturbing Agents

Use of agents known to alter the microbiome (eg, antibiotic and immunosuppressant medications) together with PPIs did not reveal additive interactions (eg, antibiotics: relative excess risk of interaction, 0.61; 95% CI, −0.56 to 1.78; *P* = .55) (Table 3; eTable 6 in Supplement 1). Among these agents, antibiotics (aIRR, 2.78; 95% CI, 2.14-3.59) and laxatives (aIRR, 2.26; 95% CI, 1.73-2.94) by themselves yielded a more than 2-fold increase in the risk of acquiring ESBL- or carbapenemase-producing Enterobacterales (adjusted for age, sex, BMI, CCI score, and simultaneous use of other agents).

**Table 3.** Incidence Rate Ratios for the Interaction Between Potential Microbiota-Disturbing Agents and ESBL- or Carbapenemase-Producing Enterobacterales Acquisition and the Risk of Interaction With PPI Use

Drug class	IRR (95% CI)		RERI (95% CI)	*P* value for interaction^b^
	Unadjusted	Adjusted^a^		
Laxative	2.90 (2.28 to 3.69)	2.26 (1.73 to 2.94)	−0.21 (−1.34 to 0.93)	.45
Immunomodulator	1.51 (1.19 to 1.92)	0.91 (0.69 to 1.20)	0.51 (−0.15 to 1.19)	.30
Antibiotic	3.29 (2.59 to 4.18)	2.78 (2.14 to 3.59)	0.61 (−0.56 to 1.78)	.55
Metformin	1.50 (0.60 to 2.34)	1.12 (0.69 to 1.82)	−0.28 (−1.61 to 1.06)	.54

Abbreviations: ESBL, extended-spectrum β-lactamase; IRR, incidence rate ratio; PPI, proton pump inhibitor; RERI, relative excess risk of interaction.

^a^ Adjusted for sex, body mass index, Charlson Comorbidity Index score, and use of antibiotics, proton pump inhibitors, immunosuppressive agents, laxatives, and metformin minus the medication of interest.

^b^ *P* value for interaction on the multiplicative scale.

### Sensitivity and Bias Analyses

The results of the sensitivity analyses are summarized in Table 4. These analyses corroborated the results of the primary analysis (eFigure 4 and eTables 7-11 in Supplement 1). To test whether our decision to not match patients based on type of specimen or bacterial species altered the results, we also adjusted for these factors in our models; the associations persisted. When we set the time risk window to 90 days, the risks remained unchanged. Excluding patients admitted from another health care facility yielded risks that were slightly higher for patients admitted from the community, but these risks had wider 95% CIs (eg, PPI use overall at 30 days: aIRR, 1.51; 95% CI, 1.16-1.97).

**Table 4.** Results of Sensitivity Analyses

Analysis	Adjusted IRR (95% CI)^a^	
	30-d risk period	90-d risk period
Secondary 1:1 matching-pairs case-control analysis	2.96 (1.14-7.74)^b^	NA
Negative control exposure (bisphosphonates or diuretics)^c^	1.35 (0.83-2.20)^d^	1.35 (0.88-2.07)^e^
**Excluding patients admitted from another health care facility**		
PPI use		
Overall^f^	1.51 (1.16-1.97)	1.51 (1.16-1.96)
Once daily	1.46 (1.10-1.95)	1.44 (1.09-1.91)
Twice daily	1.81 (1.05-3.20)	1.89 (1.14-3.14)
**Excluding patients with missing values (complete-case analysis)**		
PPI use		
Overall^f^	1.54 (1.16-2.03)	1.51 (1.15-1.98)
Once daily	1.46 (1.08-1.97)	1.41 (1.05-1.90)
Twice daily	1.99 (1.13-3.52)	1.99 (1.17-3.37)
**Restricted to patients with infection or fecal carriage^g^**		
Infection (proxy)	2.14 (1.03-4.45)	2.46 (1.18-5.13)
Fecal carriage	1.48 (1.01-2.18)	1.57 (1.06-2.33)

Abbreviations: IRR, incidence rate ratio; NA, not applicable; PPI, proton pump inhibitor.

^a^ The IRRs of the sensitivity analyses were calculated using multiple conditional logistic regression models adjusted for sex, body mass index, Charlson Comorbidity Index score, inflammatory bowel disease, and total length of intensive care unit stay. For the restricted analyses, conventional logistic regression models were used (and compared with conditional models).

^b^ Adjusted for age, sex, body mass index, Charlson Comorbidity Index score, inflammatory bowel disease, total length of intensive care unit stay, and previous solid organ or stem cell transplant.

^c^ Bisphosphonates and diuretics were identified through Anatomical Therapeutic Chemical Classification System codes M05BA and C03AA, respectively.

^d^ Includes 25 of 374 patients (6.7%) in the case group and 84 of 1865 patients (4.5%) in the control group.

^e^ Includes 32 of 374 patients (8.6%) in the case group and 111 of 1865 patients (6.0%) in the control group.

^f^ Overall exposure to PPIs and histamine 2 receptor antagonists.

^g^ Restricted to case patients (n = 52) and control patients (n = 608) with positive cultures from normally sterile sites (blood, ascites, and/or other sterile fluids) on the index date (as a proxy of infection) and patients with fecal cultures (181 patients in the case group and 466 matched patients in the control group).

We found an approximately 3-fold increased risk in the secondary case-control study, albeit with wide 95% CIs (at 30 days: aIRR, 2.96; 95% CI, 1.14-7.74). Use of thiazide diuretic or bisphosphonate medications, which we used as negative control agents, was not associated with acquisition of ESBL- or carbapenemase-producing Enterobacterales. Complete-case analyses yielded risk estimates that were consistent with the analyses after multiple imputation. Restricting the analysis to (1) the patients with ESBL- and carbapenemase-producing Enterobacterales cultured from normally sterile fluids (at 30 days: aIRR, 2.14; 95% CI, 1.03-4.45) and (2) the patients in the case and matched control groups with tested fecal swabs (at 30 days: aIRR, 1.48; 95% CI, 1.01-2.18) also yielded a slightly increased risk. The IRRs corrected for possible misclassification of patients in the control group were marginally higher than the original IRRs (eg, bias-adjusted IRRs assuming 5% and 10% of patients in the control group without fecal cultures might have had carriage were 1.75 and 1.79, respectively) (eTable 12 in Supplement 1).

## Discussion

In this nested case-control study of adult hospitalized patients, after carefully controlling for possible confounding factors, we found that PPI users had a nearly 50% increased risk (aIRR, 1.48) of acquiring ESBL- or carbapenemase-producing Enterobacterales. The risk estimate was slightly higher for twice-daily dosing compared with once-daily dosing but had wide 95% CIs. Our findings were confirmed in a second study of prospectively enrolled patients and supported by consistent results across sensitivity analyses.

A recent systematic review and meta-analysis^6^ summarized the association of PPI use with the risk of colonization with ESBL- or carbapenemase-producing Enterobacterales. Previous studies^6,36^ found that PPI use was associated with an increased risk of enteric colonization or urinary tract infection with multidrug-resistant bacteria. However, the time windows of exposure differed in the various studies,^6,36^ and most of these studies only partially corrected for possible confounding directly pertaining to PPI use.

The present study further corroborates the risk associated with PPI use found in earlier studies included in a meta-analysis.^6^ A dose-response association would provide greater evidence suggesting a potential causal relationship, but dose response could not be confirmed in the present study. However, results of a recent animal study by Woodward et al^12^ provide further information about the potential mechanism underlying the association between PPI use and increased risk of acquiring drug-resistant bacteria. The study found that increasing the intragastric pH level facilitated enteral colonization by exogenous strains.^12^

Confounding factors, such as unhealthy lifestyle or disease severity, among PPI users could produce overestimation of the risk found in previous studies.^6,28^ Adjustment for proxies of disease severity (CCI score, surgical procedures, length of stay in the ICU, and hospitalization days in the past 6 months) slightly attenuated our unadjusted estimate of the IRR for acquisition of ESBL- or carbapenemase-producing Enterobacterales without negating the risk. These variables are known to be associated with the likelihood of PPI use^7,37^ and may be surrogate markers of frequent exposure to antibiotics. Further control for markers of disease severity or factors that reflected differences associated with PPI treatment did not appreciably change the adjusted estimates, suggesting we minimized confounding by disease severity.

Across subgroups, we observed consistently higher risk of acquisition for factors that might alter the baseline risk of colonization or infection in the studied population (eg, CCI score >2 and cancer diagnosis as alternative markers of immunosuppression). There was no evidence of an additive interaction between the use of PPIs and other agents that have been reported as strongly associated with microbiome dysbiosis.^25^

The risk factors associated with acquisition of ESBL- or carbapenemase-producing Enterobacterales identified in the study population were consistent with well-known factors reported in previous studies^30,38^ and further strengthened the validity of our findings. Notably, laxatives yielded a higher risk than previously reported.^39^ Similar to antibiotics,^40^ it is possible that the positive association of laxatives with acquisition of ESBL- or carbapenemase-producing Enterobacterales is mediated via their disturbance of the microbiome. The risk among patients with probable infection was similar to that of patients who were only colonized, which suggests that PPI use probably does not increase the risk of infection.

### Strengths and Limitations

This study has several strengths, including the focus on careful correction for confounding factors and confirmation of the findings in a secondary prospectively conducted case-control study. We used a directed acyclic graph to visualize the complex interactions of study factors to dissect possible confounding bias. In addition, we conducted several sensitivity analyses and used a negative-control exposure to corroborate our results.

The study also has limitations. First, despite these careful analyses, there remains the possibility of unmeasured confounding. The measures we took to circumvent this possibility (including the design with patients in case and control groups matched for age, the risk-set sampling of patients in the control group, and the sensitivity analyses, negative-control exposure, and secondary case-control study) provide confidence that our results are not mainly due to uncontrolled confounding. Second, the analyses were based on prevalent rather than incident use of PPIs, which could introduce prevalent-user bias. Selection bias of participants in the control group is unlikely because we used a nested design in a defined same-source population of admitted patients.^20^ Patients in both the case and control groups were selected based on clinical cultures to enhance group comparability.

Third, bias from misclassification of the exposure is unlikely given the use of accurate hospital pharmacy data; medication verification at admission is standard practice at our centers. In addition, the potential adverse effect of PPIs investigated in this study was not well known at the time we performed the study,^41^ and the retrospective design prevented possible bias from foreknowledge of the study’s purpose during sampling and data collection. Fourth, we used clinical cultures instead of screening cultures to assess the presence or absence of ESBL- and/or carbapenemase-producing strains, which could lead to misclassification of patients in the control group, in whom carriage could go undetected. However, misclassification of those in the control group often leads to dilution of risk estimates. Hence, the 1.48-fold increased risk of acquiring drug-resistant strains among patients using PPIs is, at worst, a low estimate of the true risk. We confirmed this finding in a misclassification analysis, which revealed that the risk estimate slightly increased when correcting for misclassified patients in the control group.

Fifth, although the setting comprised 2 tertiary care hospitals, we specifically designed this study to measure associations of exposure to PPIs by carefully controlling for confounding bias, and we found an IRR that was close to the pooled risk (OR, 1.60; 95% CI, 1.33-1.92) reported in the previous meta-analysis.^6^ Of course, the risks observed in this study apply to a hospitalized patient population. Sixth, an insufficient number of patients who used higher doses of PPIs and histamine 2 receptor antagonists precluded the ability to reach definitive conclusions about dose response.

While a randomized clinical trial could overcome bias related to nonrandomization, such a trial would be limited by ethical and feasibility constraints. A previous large trial^42^ examining the safety of PPI treatment provided no information on drug-resistant organisms, although a higher incidence of enteric infections among PPI users was noted. This higher incidence can be considered a surrogate marker of introduction of exogenous microorganisms.^42^

## Conclusions

The findings of this case-control study support the role of PPI use as an independent risk factor associated with the acquisition of ESBL- or carbapenemase-producing Enterobacterales. Given that PPIs are widely misused, judicious use of PPIs is warranted to potentially prevent the associated acquisition of ESBL- or carbapenemase-producing Enterobacterales.

## Notes

Supplement 1. **eMethods 1.** Microbiological Analysis, Clinical Data Extraction, Directed Acyclic Graph, Calculation of Relative Excess Risk Due to Interaction, Precision, Secondary Case-Control Analysis, and Multiple Imputation **eMethods 2.** R Code for the Directed Acyclic Graph **eTable 1.** *ICD-10* and ATC Codes **eTable 2.** Analysis of Potential Risk Factors for Acquisition of ESBL- or Carbapenemase-Producing Enterobacterales in the 1:5 Matched Case-Control Population **eTable 3.** Analyses of the Acquisition of ESBL- or Carbapenemase-Producing Enterobacterales With Individual Antibiotics **eTable 4.** Distribution of ESBL- and Carbapenemase-Producing Organisms in the Case Group **eTable 5.** Stratified Analyses for the Acquisition of ESBL- or Carbapenemase-Producing Enterobacterales (Interaction With PPI Use) **eTable 6.** Additive Interaction Between Potential Microbiota-Disturbing Agents and PPI Treatment in Determining Acquisition of ESBL- or Carbapenemase-Producing Enterobacterales **eTable 7.** Analyses of the Acquisition of ESBL- or Carbapenemase-Producing Enterobacterales After Excluding Patients Admitted From Another Health Care Facility **eTable 8.** Analyses of the Acquisition of ESBL- or Carbapenemase-Producing Enterobacterales After Excluding Patients With Missing Values (Complete-Case Analysis) **eTable 9.** Analyses of the Acquisition of ESBL- or Carbapenemase-Producing Enterobacterales With the Negative Control Exposure **eTable 10.** Characteristics and Analysis of the 1:1 Matching-Pairs Population **eTable 11.** Analyses of the Acquisition of ESBL- or Carbapenemase-Producing Enterobacterales in the 1:1 Matching-Pairs Case-Control Study **eTable 12.** Analysis of Misclassification **eFigure 1.** Visualization of the Study Design and Time Frame **eFigure 2.** Directed Acyclic Graph **eFigure 3.** Flow Diagram of the Nested 1:5 Matched Design **eFigure 4.** Flow Diagram of the 1:1 Matching-Pairs Study (Prospectively Enrolled)
eReferences

Supplement 2.
Data Sharing Statement
