# Supplementary material for: Association of Proton Pump Inhibitor Use With Risk of Acquiring Drug-Resistant Enterobacterales
Source: JAMA Netw Open. 2023 Feb 23;6(2):e230470. doi: 10.1001/jamanetworkopen.2023.0470 (PMC9951039; doi:10.1001/jamanetworkopen.2023.0470)
Supplement: Supplement 1. — eMethods 1. Microbiological Analysis, Clinical Data Extraction, Directed Acyclic Graph, Calculation of Relative Excess Risk Due to Interaction, Precision, Secondary Case-Control Analysis, and Multiple Imputation eMethods 2. R Code for the Directed Acyclic Graph eTable 1. ICD-10 and ATC Codes eTable 2. Analysis of Potential Risk Factors for Acquisition of ESBL- or Carbapenemase-Producing Enterobacterales in the 1:5 Matched Case-Control Population eTable 3. Analyses of the Acquisition of ESBL- or Carbapenemase-Producing Enterobacterales With Individual Antibiotics eTable 4. Distribution of ESBL- and Carbapenemase-Producing Organisms in the Case Group eTable 5. Stratified Analyses for the Acquisition of ESBL- or Carbapenemase-Producing Enterobacterales (Interaction With PPI Use) eTable 6. Additive Interaction Between Potential Microbiota-Disturbing Agents and PPI Treatment in Determining Acquisition of ESBL- or Carbapenemase-Producing Enterobacterales eTable 7. Analyses of the Acquisition of ESBL- or Carbapenemase-Producing Enterobacterales After Excluding Patients Admitted From Another Health Care Facility eTable 8. Analyses of the Acquisition of ESBL- or Carbapenemase-Producing Enterobacterales After Excluding Patients With Missing Values (Complete-Case Analysis) eTable 9. Analyses of the Acquisition of ESBL- or Carbapenemase-Producing Enterobacterales With the Negative Control Exposure eTable 10. Characteristics and Analysis of the 1:1 Matching-Pairs Population eTable 11. Analyses of the Acquisition of ESBL- or Carbapenemase-Producing Enterobacterales in the 1:1 Matching-Pairs Case-Control Study eTable 12. Analysis of Misclassification eFigure 1. Visualization of the Study Design and Time Frame eFigure 2. Directed Acyclic Graph eFigure 3. Flow Diagram of the Nested 1:5 Matched Design eFigure 4. Flow Diagram of the 1:1 Matching-Pairs Study (Prospectively Enrolled) eReferences [file jamanetwopen-e230470-s001.pdf]

Supplementary Online Content

Willems RPJ, Schut MC, Kaiser AM, et al. Association of proton pump inhibitor use with risk of acquiring drug-resistant Enterobacterales. *JAMA Netw Open*. 2023;6(2):e230470. doi:10.1001/jamanetworkopen.2023.0470

eMethods 1. Microbiological Analysis, Clinical Data Extraction, Directed Acyclic Graph, Calculation of Relative Excess Risk Due to Interaction, Precision, Secondary Case-Control Analysis, and Multiple Imputation

eMethods 2. R Code for the Directed Acyclic Graph

eTable 1. *ICD-10* and ATC Codes

eTable 2. Analysis of Potential Risk Factors for Acquisition of ESBL- or Carbapenemase-Producing Enterobacterales in the 1:5 Matched Case-Control Population

eTable 3. Analyses of the Acquisition of ESBL- or Carbapenemase-Producing Enterobacterales With Individual Antibiotics

eTable 4. Distribution of ESBL- and Carbapenemase-Producing Organisms in the Case Group

eTable 5. Stratified Analyses for the Acquisition of ESBL- or Carbapenemase-Producing Enterobacterales (Interaction With PPI Use)

eTable 6. Additive Interaction Between Potential Microbiota-Disturbing Agents and PPI Treatment in Determining Acquisition of ESBL- or Carbapenemase-Producing Enterobacterales

eTable 7. Analyses of the Acquisition of ESBL- or Carbapenemase-Producing Enterobacterales After Excluding Patients Admitted From Another Health Care Facility

eTable 8. Analyses of the Acquisition of ESBL- or Carbapenemase-Producing Enterobacterales After Excluding Patients With Missing Values (Complete-Case Analysis)

eTable 9. Analyses of the Acquisition of ESBL- or Carbapenemase-Producing Enterobacterales With the Negative Control Exposure

eTable 10. Characteristics and Analysis of the 1:1 Matching-Pairs Population

eTable 11. Analyses of the Acquisition of ESBL- or Carbapenemase-Producing Enterobacterales in the 1:1 Matching-Pairs Case-Control Study

eTable 12. Analysis of Misclassification

eFigure 1. Visualization of the Study Design and Time Frame

eFigure 2. Directed Acyclic Graph

eFigure 3. Flow Diagram of the Nested 1:5 Matched Design

eFigure 4. Flow Diagram of the 1:1 Matching-Pairs Study (Prospectively Enrolled)

eReferences

This supplemental material has been provided by the authors to give readers additional information about their work.

© 2023 Willems RPJ et al. *JAMA Network Open*.

eMethods 1. Microbiological Analysis, Clinical Data Extraction, Directed Acyclic Graph, Calculation of Relative Excess Risk Due to Interaction, Precision, Secondary Case-Control Analysis, and Multiple Imputation

Microbiological Analysis

Cases were identified from the Clinical Microbiological Laboratory Information System (CMLIS), which included patients yielding newly-detected extended-spectrum β-lactamase and/or carbapenemase producing Enterobacterales (ESBL-E/CPE) isolates in select clinical specimens. ESBL-E/CPE acquisition was defined by a positive culture after microbiological laboratory testing of specimens submitted during hospitalization at Amsterdam UMC (multidrug resistance was defined according to Magiorakis *et al*.)[1] In addition, hospital policy dictates screening for multidrug-resistant Gram-negative bacteria (MDR-GNB) in high-risk patients using the following criteria: (1) previous admission to a foreign hospital; (2) residents of centres for asylum seekers; (3) active livestock farming; (4) dialysis in a foreign hospital; (5) as part of contact investigation; (6) known carriers; (7) outbreak in another hospital; (8) at and during admission to the haematology and intensive care units. Culture-positive test results were confirmed by the clinical microbiologic laboratory compliant with European Committee on Antimicrobial Susceptibility Testing (EUCAST) guidelines.[2] ESBL-E/CPE were detected via standard culture methods according to the manufacturer's instructions. Specimens eligible for inclusion were: faeces, urine, blood, sputum and ascites. Species identification was performed using the VITEK-MS system (bioMérieux, Marcy-l'Etoile, France) and antibiotic susceptibility testing was done with the VITEK 2 system (bioMérieux, Marcy-l'Etoile, France). ESBL production was phenotypically confirmed using the combination disc test. Carbapenemase activity was confirmed in isolates with a VITEK-MIC for meropenem of ≥0.25 mg/L by using a meropenem E-test (bioMérieux), the CIM test and the inhibition tests (Rosco, Taastrup, Denmark).[3] Genotypic analysis of ESBL producers was not part of standard work-up. Genetic analysis of carbapenemase-producing isolates was performed with multiplex polymerase chain-reaction and sequencing.

Clinical Data Extraction

Data items were extracted from the Amsterdam UMC Research Data Platform (RDP). The Research Data Platform (RDP) is a de-identified clinical research data warehouse that includes data recorded prospectively from standard care, facilitating the construction of integrated data sets; clusters of data are extracted from the hospital electronic medical records (e.g. medical diagnoses) and other sources (e.g. prescriptions). This platform is subject to audits and compliant with privacy regulations. Data are updated in an ongoing basis via the linkage of several data sources.

Directed Acyclic Graph

We used a directed acyclic graph (DAG) to determine confounding factors (DAGitty v3.0).[4, 5] Alternative assumptions were considered; these were judged and extensively discussed by all authors to arrive at the final DAG to inform the analyses (eFigure 3). Covariates encoded in the DAG were based on the literature.[6-9]

Calculation of Relative Excess Risk Due to Interaction

Additive interaction was assessed using the relative excess risk due to interaction (RERI) as calculated by:[10]

$$RERI = RR_{11} - RR_{10} - RR_{01} + 1$$

where RR_{11} indicates the relative risk of the outcome when both factors are present; RR_{10} indicates the relative risk of the outcome when only the first factor is present; and, RR_{01} indicates the relative risk of the outcome when only the second factor is present. A RERI >0 implies positive interaction.

© 2023 Willems RPJ et al. *JAMA Network Open*.

Precision

We determined expected precision in the study design phase. Using a 1:5 set matching ratio and assuming a study size of approximately 2400 (390 cases and 1960 controls) patients, we conducted an exploratory precision analysis.[11] This could yield an OR of 2.00 for ESBL-E/CPE acquisition with a level of precision of 1.55 (ratio of upper to lower confidence interval). The parameters were derived from a previous Dutch study.[12]

Secondary Case-Control Analysis

A separate case-control study was conducted in order to compare the findings from the primary 1:5 matched nested design and assess for a set of residual confounders using survey data. The in-, exclusion and matching criteria were similar to the retrospective study. Consecutive cases were enrolled prospectively between March 21, 2019 and July 19, 2021, and individually matched (1:1) to a control patient. We used pair-matching with calipers for greedy matching (without replacement).

Clinical data was extracted from the hospital' database (RDP). A questionnaire was administered to collect data on information not recorded in hospital records. Questionnaires were processed using specific collection software,[13] collecting potential unmeasured confounding factors between the use of proton pump inhibitors and acquisition of ESBL-E/CPE: ethnicity (Western vs non-Western), body mass index (<25 vs 25-30 vs ≥30 kg/m^2), alcohol use (no intake vs any), smoking status (current smoker vs non-smoker), hospitalization within the previous 6 months (yes vs no), and international travel within the previous 6 months (yes vs no). A total of 188 patients (94 cases and 94 controls) were analyzed.

Multiple Imputation

To impute (continuous) missing values for body mass index (26%), we used multiple imputation (MICE) with 20 replications based on the outcome and all variables included in the analysis, combined using Rubin's rules.[14-17] For imputation, we used predictive mean matching (5 nearest neighbours) to safeguard against implausible predictions. Convergence was determined graphically. For missing body mass index values, we assigned values from the proximal year if available and imputed missing values for the remaining values. Sensitivity was assessed by comparison with a complete-case analysis.

© 2023 Willems RPJ et al. *JAMA Network Open*.

eMethods 2. R Code for the Directed Acyclic Graph

```
testImplications <- function( covariance.matrix, sample.size ){
		library(ggm)
		tst <- function(i){ pcor.test( pcor(i,covariance.matrix), length(i)-2, sample.size )$pvalue }
tos <- function(i){ paste(i,collapse=" ") }
implications <- list(c("Admission from healthcare facility","Antibiotic exposure","ICU stay","Comorbidity","LOS","Transplantation"),
		c("Admission from healthcare facility","Antibiotic exposure","Age","Comorbidity","Frailty"),
		c("Admission from healthcare facility","BMI proxy","Comorbidity","Age","Frailty"),
		c("Admission from healthcare facility","Bacterial species","Comorbidity","Frailty","Age"),
		c("Admission from healthcare facility","ICU stay","Age","Comorbidity"),
		c("Admission from healthcare facility","Inflammatory bowel disease"),
		c("Admission from healthcare facility","Medical device use","Age","Comorbidity","Frailty"),
		c("Admission from healthcare facility","PPI use","Age","Comorbidity","Sex","BMI proxy"),
		c("Admission from healthcare facility","PPI use","Comorbidity","Age","Frailty"),
		c("Admission from healthcare facility","Comedication","Comorbidity","LOS"),
		c("Admission from healthcare facility","Comedication","Age","Comorbidity","Frailty"),
		c("Admission from healthcare facility","Immunosuppressants","Comorbidity","LOS","Transplantation"),
		c("Admission from healthcare facility","Immunosuppressants","Age","Comorbidity","Frailty"),
		c("Admission from healthcare facility","LOS","Age","Comorbidity","Frailty"),
		c("Admission from healthcare facility","Sex","Age","Comorbidity","Frailty"),
		c("Admission from healthcare facility","Surgery","Age","Comorbidity"),
		c("Admission from healthcare facility","Transplantation","Comorbidity"),
		c("Antibiotic exposure","BMI proxy","LOS","ICU stay","Comorbidity","Transplantation"),
		c("Antibiotic exposure","Inflammatory bowel disease"),
		c("Antibiotic exposure","Medical device use","Comorbidity","LOS","ICU stay","Transplantation"),
		c("Antibiotic exposure","PPI use","Age","Comorbidity","Sex","BMI proxy","ICU stay"),
		c("Antibiotic exposure","PPI use","LOS","ICU stay","Comorbidity","Transplantation"),
		c("Antibiotic exposure","Age","Comorbidity","ICU stay","Transplantation","LOS"),
		c("Antibiotic exposure","Comedication","Comorbidity","LOS"),
		c("Antibiotic exposure","Immunosuppressants","Comorbidity","LOS","Transplantation"),
		c("Antibiotic exposure","Sex","LOS","ICU stay","Comorbidity","Transplantation"),
		c("Antibiotic exposure","Surgery","Age","Comorbidity"),
		c("Antibiotic exposure","Surgery","Comorbidity","ICU stay","Transplantation","LOS"),
		c("Antibiotic exposure","Frailty","Comorbidity","ICU stay","Transplantation","LOS"),
		c("BMI proxy","ICU stay","Age","Comorbidity"),
		c("BMI proxy","Inflammatory bowel disease"),
		c("BMI proxy","Comedication","Comorbidity","LOS"),
		c("BMI proxy","Immunosuppressants","Comorbidity","LOS","Transplantation"),
		c("BMI proxy","Surgery","Age","Comorbidity"),
		c("BMI proxy","Transplantation","Comorbidity"),
		c("Bacterial species","Surgery","Age","Comorbidity"),
		c("ICU stay","Inflammatory bowel disease"),
		c("ICU stay","Comedication","Comorbidity","LOS"),
		c("ICU stay","Immunosuppressants","Comorbidity","LOS","Transplantation"),
		c("ICU stay","Sex","Comorbidity","Age"),
		c("ICU stay","Surgery","Age","Comorbidity"),
		c("ICU stay","Transplantation","Comorbidity"),
		c("ICU stay","Frailty","Age","Comorbidity"),
		c("Inflammatory bowel disease","Medical device use"),
		c("Inflammatory bowel disease","Age"),
		c("Inflammatory bowel disease","Comorbidity"),
		c("Inflammatory bowel disease","Immunosuppressants"),
		c("Inflammatory bowel disease","LOS"),
		c("Inflammatory bowel disease","Sex"),
		c("Inflammatory bowel disease","Surgery"),
		c("Inflammatory bowel disease","Transplantation"),
		c("Inflammatory bowel disease","Frailty"),
		c("Medical device use","PPI use","Age","Comorbidity","Sex","BMI proxy","ICU stay"),
		c("Medical device use","Comedication","Comorbidity","LOS"),
		c("Medical device use","Immunosuppressants","Comorbidity","LOS","Transplantation"),
		c("Medical device use","Surgery","Age","Comorbidity"),
		c("PPI use","Comedication","Inflammatory bowel disease","Comorbidity","LOS"),
		c("PPI use","Comedication","Sex","Comorbidity","Age","BMI proxy","ICU stay","Inflammatory bowel disease"),
		c("PPI use","Immunosuppressants","Comorbidity","LOS","Transplantation"),
		c("PPI use","Immunosuppressants","Sex","Comorbidity","Age","BMI proxy","ICU stay"),
		c("PPI use","LOS","Sex","Comorbidity","Age","BMI proxy","ICU stay"),
		c("PPI use","Surgery","Age","Comorbidity"),
		c("PPI use","Transplantation","Comorbidity"),
		c("PPI use","Frailty","Age","Sex","Comorbidity","BMI proxy"),
		c("Age","Comedication","Comorbidity","LOS"),
		c("Age","Immunosuppressants","Comorbidity","LOS","Transplantation"),
		c("Age","Sex"),
```

© 2023 Willems RPJ et al. *JAMA Network Open*.

```
                    c("Age","Transplantation","Comorbidity"),
                    c("Comedication","Immunosuppressants","Comorbidity","LOS"),
                    c("Comedication","Sex","LOS","Comorbidity"),
                    c("Comedication","Surgery","Age","Comorbidity"),
                    c("Comedication","Surgery","Comorbidity","LOS"),
                    c("Comedication","Transplantation","Comorbidity","LOS"),
                    c("Comedication","Frailty","Comorbidity","LOS"),
                    c("Immunosuppressants","Sex","LOS","Comorbidity","Transplantation"),
                    c("Immunosuppressants","Surgery","Age","Comorbidity"),
                    c("Immunosuppressants","Surgery","Comorbidity","Transplantation","LOS"),
                    c("Immunosuppressants","Frailty","Comorbidity","Transplantation","LOS"),
                    c("LOS","Surgery","Age","Comorbidity"),
                    c("Sex","Surgery","Age","Comorbidity"),
                    c("Sex","Transplantation","Comorbidity"),
                    c("Surgery","Transplantation","Comorbidity"),
                    c("Surgery","Frailty","Age","Comorbidity"),
                    c("Transplantation","Frailty","Comorbidity"))
          data.frame( implication=unlist(lapply(implications,tos)),
                    pvalue=unlist( lapply( implications, tst ) ) )
}
```

eTable 1. *ICD-10* and ATC Codes

A. *ICD-10* Codes

Comorbidities[a]	Score	ICD-10 code
Inflammatory bowel disease	0	K50.0, K50.1, K50.8,
Myocardial infarction	1	I21, I22, I23
Congestive heart failure	1	I50, I11.0, I13.0, I13.2
Peripheral vascular disease	1	I70, I71, I72, I73, I74, I77
Cerebrovascular disease	1	I60-I69, G45, G46
Dementia	1	F00-F03, F05.1, G30
Chronic pulmonary disease	1	J40, JJ45-47, J60-J67, J68.4, J70.1, J70.3, J84.1, J92.0, J96.1, J98.2, J98.3
Connective tissue disease	1	M05, M06, M08, M09, M30, M31, M32, M33, M34, M35, M36, D86
Ulcer disease	1	K22.1, K25-K28
Mild liver disease	1	B18, K70.0-K70.3, K70.9, K71, K73, K74, K76.0
Diabetes types 1 and 2	1	E10.0, E10.1, E10.9, E11.0, E11.1, E11.9
Hemiplegia	2	G81, G82
Moderate to severe kidney disease	2	12, I13, N00-N05, N07, N11, N14, N17-N19, Q61
Diabetes with end-organ damage	2	E10.2-E10.8, E11.2-E11.8
Any tumor	2	C00-C75
Leukemia	2	C91-C95
Lymphoma	2	C81-C85, C88, C90, C96
Moderate to severe liver disease	3	B15.0, B16.0, B16.2, B19.0, K70.4, K72, K76.6, I85
Metastatic solid tumor	6	C76-C80
AIDS	6	B21-B24

Other covariates	Description
Prior hospitalization	Prior admission within 6 months before the index. A 14-day time gap was used to distinguish the admission in which the culture (index) was detected from previous hospital admissions.
ICU admission	Prior length of stay in the ICU within 90 days before the index.
Surgery	Defined according to in-hospital surgical procedure codes for abdominal (including endoscopic retrograde cholangiopancreatography), urogenital and/or cardiovascular surgery within the 90 days before the index.
Transplantation	Defined according to in-hospital transplantation codes for solid organ transplantation and/or stem cell transplantation within 90 days before the index.
Admission from another healthcare facility	Patients admitted from a hospital abroad or patients admitted another Dutch hospital, rehabilitation clinic or long-term care facility.
Katz (ADL) index	The measurement proximal before the index was used.

ADL, activities of daily living; ICD-10, International Classification of Diseases, Tenth Revision; ICU, intensive care unit.
[a] Charlson Comorbidity Index scores were calculated using the R (R Foundation for Statistical Computing) 'comorbidity' package (Gasparini et al. 2018).

© 2023 Willems RPJ et al. *JAMA Network Open*.

B. ATC Codes

Drug class[a]	ATC classification system codes
Immunosuppressive agents	L01, L04, and H02AB; D07; R03BA.
Antirheumatic agents	M01A; M01BA; M01C
Proton pump inhibitors[b]	A02BC: A02BC01, A02BC02, A02BC03, A02BC04, A02BC05, A02BC06, A02BC07, A02BC53, A02BC54
Histamine-2 receptor antagonists	A02BA: A02BA01, A02BA02, A02BA03, A02BA04, A02BA05, A02BA06, A02BA07, A02BA08, A02BA51, A02BA53
Antacids[c]	A02A
Laxatives	A06AA, A06AB, A06AC, A06AD
Metformin	A10BA02
Broad-spectrum antibiotics	J01AA, J01CA, J01CR, J01DB, J01DC, J01DD, J01DH, J01EE, J01GB, J01MA, and J01XB
Narrow-spectrum antibiotics	J01BA, J01CE, J01CF, J01DE, J01DF, J01EA, J01EB, J01FA, J01FF, J01XA, J01XC, J01XD, J01XE, and J01XX
Penicillin with extended spectrum	J01CA
Penicillins with small spectrum	J01CE and J01CF
Trimethoprim-sulfamethoxazole	J01EE, J01EA, J01EB
Macrolides	J01F
Nitrofurantoin	J01XE01
Fluoroquinolones	J01MA
Cephalosporins	J01DB, J01DC, J01DD, J01DE
**Other covariates**	**Description**
Antibiotic days	Defined as the cumulative number of in-hospital days exposed to the individual antibiotic until the date of the culture or antibiotic suspension, whichever came earlier.

[a] Use of medication refers to the 30 days before the index.
[b] >70% of ATC codes on the patient-level corresponded to pantoprazole sodium with approximately 25-30% omeprazole/esomeprazole; less than 1% receiving lansoprazole/rabeprazole.
[c] The few numbers of users of antacids precluded analysis of these medications.

© 2023 Willems RPJ et al. *JAMA Network Open*.

eTable 2. Analysis of Potential Risk Factors for Acquisition of ESBL- or Carbapenemase-Producing Enterobacterales in the 1:5 Matched Case-Control Population[a]

Characteristic	Sex-adjusted IRR (95%CI)	Sex, and CCI-adjusted IRR (95% CI)	Sex, BMI, CCI, and medication-adjusted IRR (95%CI)[c]
Age at index date, y	NA	NA	
Female	0.98 (0.78-1.22)	1.03 (0.82-1.29)	
Admitted from another healthcare facility	1.46 (1.01-2.11)	1.60 (1.10-2.32)	
BMI (kg/m^2)			
<25	1 [Reference]	1 [Reference]	
25-30	0.89 (0.66-1.20)[b]	0.94 (0.69-1.27)[b]	
≥30	1.13 (0.82-1.56)[b]	1.18 (0.86-1.64)[b]	
CCI score	1.10 (1.06-1.15)	1.10 (1.06-1.15)	
0	1 [Reference]	1 [Reference]	
1-2	1.59 (1.18-2.14)	1.59 (1.18-2.14)	
>2	2.58 (1.93-3.46)	2.58 (1.93-3.46)	
Coexisting disease			
Myocardial infarction	1.67 (1.14-2.44)	1.46 (0.99-2.15)	
Congestive heart failure	2.63 (1.89-3.65)	2.24 (1.60-3.14)	
Peripheral vascular disease	1.72 (1.16-2.56)	1.47 (0.98-2.19)	
Neurologic disease	1.59 (1.07-2.35)	1.37 (0.92-2.04)	
Chronic pulmonary disease	0.97 (0.66-1.43)	0.81 (0.55-1.22)	
Rheumatologic disease	1.05 (0.59-1.89)	1.06 (0.59-1.91)	
Peptic ulcer disease	1.66 (0.54-5.15)	1.29 (0.41-4.07)	
Liver disease	2.85 (1.77-4.58)	2.25 (1.38-3.68)	
Diabetes mellitus	2.03 (1.51-2.73)	1.69 (1.23-2.30)	
Kidney disease	2.68 (2.03-2.53)	2.27 (1.68-3.06)	
Cancer	1.18 (0.91-1.52)	0.67 (0.47-0.93)	
Inflammatory bowel disease	1.36 (0.87-2.13)	1.27 (0.81-2.00)	
Katz (ADL) index	0.91 (0.83-0.99)	0.93 (0.85-1.02)	
Prior admission to ICU	3.22 (2.10-4.94)	3.12 (2.03-4.81)	
Days in the ICU	1.15 (1.08-1.22)	1.14 (1.07-1.21)	
Surgery	2.15 (1.60-2.88)	1.87 (1.39-2.54)	
Transplant	2.57 (1.62-4.08)	2.24 (1.40-3.59)	
Medication			
Antirheumatic drug	1.12 (0.71-1.79)	1.04 (0.65-1.67)	
Metformin	1.50 (0.60-2.34)	1.26 (0.80-1.98)	1.12 (0.69-1.82)
Laxative	2.90 (2.28-3.69)	2.66 (2.08-3.41)	2.26 (1.73-2.94)
Immunomodulating drug	1.51 (1.19-1.92)	1.32 (1.03-1.70)	0.91 (0.69-1.20)
Antibiotic (≥1)	3.29 (2.59-4.18)	3.12 (2.45-3.97)	2.78 (2.14-3.59)
PPI only	1.73 (1.36-2.21)	1.51 (1.17-1.95)	

ADL, activities of daily living; BMI, body mass index; CCI, Charlson Comorbidity Index; H2RA, histamine-2 receptor antagonist; ICU, intensive care unit; IRR, incidence rate ratios; PPI, proton pump inhibitor.

[a] Cases and controls were matched on age and the date the case tested positive (±15 days).

[b] Includes imputed values. For individuals with missing BMI values at the index, we assigned values from the proximal year if available and imputed missing values for the remaining (581[25.9%]) using multiple imputation with chained equations.

[c] Adjusted for sex, BMI, Charlson Comorbidity Index score, use of antibiotics, proton pump inhibitors, immunosuppressant agents, laxatives, and metformin, minus medication of interest.

© 2023 Willems RPJ et al. *JAMA Network Open*.

eTable 3. Analyses of the Acquisition of ESBL- or Carbapenemase-Producing Enterobacterales With Individual Antibiotics

**Antibiotic group[a,b]**	**Controls (n = 1865)**	**Cases (n = 374)**	**Sex-adjusted IRR (95% CI)**	**Adjusted IRR (95% CI)[c]**
**Any antibiotics**				
Nonuse	1,418 (76.0%)	188 (50.3%)	1 [Reference]	1 [Reference]
Use	447 (24.0%)	186 (49.7%)	3.29 (2.59-4.18)	3.12 (2.45-3.97)
**Broad-spectrum antibiotics**			1.08 (1.06-1.10)[d]	1.06 (1.04-1.08)[d]
Nonuse	1,491 (79.9%)	212 (56.7%)	1 [Reference]	1 [Reference]
Use	374 (20.1%)	162 (43.3%)	3.17 (2.49-4.04)	3.05 (2.39-3.90)
**Narrow-spectrum antibiotics**			1.15 (1.08-1.21)[d]	1.08 (1.02-1.15)[d]
Nonuse	1,744 (93.5%)	319 (85.3%)	1 [Reference]	1 [Reference]
Use	121 (6.5%)	55 (14.7%)	2.56 (1.80-3.63)	2.24 (1.57-3.20)
**Broad-spectrum penicillin**			1.33 (1.18-1.51)[d]	1.33 (1.17-1.51)[d]
Nonuse	1,833 (98.3%)	351 (93.9%)	1 [Reference]	1 [Reference]
Use	32 (1.7%)	23 (6.1%)	3.65 (2.12-6.27)	3.69 (2.13-6.38)
**Narrow-spectrum penicillin**			1.09 (1.02-1.17)[d]	1.05 (0.98-1.13)[d]
Nonuse	1,818 (97.5%)	356 (95.2%)	1 [Reference]	1 [Reference]
Use	47 (2.5%)	18 (4.8%)	2.00 (1.13-3.51)	1.80 (1.02-3.19)
**Trimethoprim-sulfamethoxazole**			1.08 (1.04-1.11)[d]	1.06 (1.03-1.10)[d]
Nonuse	1,802 (96.6%)	332 (88.8%)	1 [Reference]	1 [Reference]
Use	63 (3.4%)	42 (11.2%)	3.61 (2.39-5.45)	3.52 (2.32-5.33)
**Macrolides**			0.83 (0.56-1.23)[d]	0.81 (0.55-1.21)[d]
Nonuse	1,826 (97.9%)	368 (98.4%)	1 [Reference]	1 [Reference]
Use	39 (2.1%)	6 (1.6%)	0.76 (0.32-1.82)	0.74 (0.31-1.77)
**Nitrofurantoin**			1.09 (1.02-1.17)[d]	1.08 (1.002-1.16)[d]
Nonuse	1,825 (97.9%)	360 (96.3%)	1 [Reference]	1 [Reference]
Use	40 (2.1%)	14 (3.7%)	1.82 (0.97-3.42)	1.80 (0.96-3.40)
**Fluoroquinolone**			1.09 (1.05-1.13)[d]	1.06 (1.02-1.10)[d]
Nonuse	1,705 (91.4%)	294 (78.6%)	1 [Reference]	1 [Reference]
Use	160 (8.6%)	80 (21.4%)	2.89 (2.15-3.90)	2.82 (2.09-3.81)
**Cephalosporin**			1.76 (1.38-2.24)[d]	1.58 (1.23-2.04)[d]
Nonuse	1,786 (95.8%)	334 (89.3%)	1 [Reference]	1 [Reference]
Use	79 (4.2%)	40 (10.7%)	2.72 (1.82-4.06)	2.80 (1.87-4.2)

IRR, incidence rate ratios.
Cases represent newly-detected colonization and/or infection with ESBL-E/CPE.
[a] IRRs were calculated using conditional logistic regression matched by age.
[b] Within the previous 30 days before the index.
[c] Conditioned on matched sets (i.e. age) and adjusted for sex (male, female), Charlson Comorbidity Index score (continuous).
[d] Adjusted IRR reflects the risk per daily increment of in-hospital exposure; additionally adjusted for time to infection.

© 2023 Willems RPJ et al. *JAMA Network Open*.

eTable 4. Distribution of ESBL- and Carbapenemase-Producing Organisms in the Case Group

**1:5 Matched case-control design**			
**Organism**	**Total**	**ESBL-producing**	**Carbapenemase-producing**
Enterobacter spp.	6 (1.6%)	6 (1.6%)	0 (0%)
Escherichia coli	274 (73.3%)	266 (75.4%)	8 (38.1%)
Klebsiella spp.	78 (20.9%)	67 (19.0%)	11 (52.4%)
Other	16 (4.3%)	14 (4.0%)	2 (9.5%)
	374 (100%)	353 (100%)	21 (100%)
**1:1 Matching-pairs case-control design**			
**Organism**	**Total**	**ESBL-producing**	**Carbapenemase-producing**
Enterobacter spp.	3 (3.2%)	3 (3.3%)	0 (0%)
Escherichia coli	70 (70.5%)	69 (75.0%)	1 (50.0%)
Klebsiella spp.	19 (20.2%)	18 (19.5%)	1 (50.0%)
Other	2 (2.1%)	2 (21.7%)	0 (0%)
	94 (100%)	92 (100%)	2 (100%)

ESBL, extended-spectrum β-lactamase.

© 2023 Willems RPJ et al. *JAMA Network Open*.

eTable 5. Stratified Analyses for the Acquisition of ESBL- or Carbapenemase-Producing Enterobacterales (Interaction With PPI Use)[a]

			**Unconditional regression analysis**[b]				**Conditional regression analysis**[b]			
**Subgroup**	**Cases (n/N)**	**Controls (n/N)**	**IRR (95% CI)**[c]	$P_{[interaction]}$	**IRR (95% CI)**[d]	$P_{[interaction]}$	**IRR (95% CI)**[c]	$P_{[interaction]}$	**IRR (95% CI)**[d]	$P_{[interaction]}$
**Overall (0-30 d)**	132/374	452/1865	1.72 (1.35-2.19)		1.46 (1.14-1.88)		1.74 (1.37-2.22)		1.48 (1.15-1.91)	
**Sex**				0.67		0.80		0.72		0.73
Male	73/193	241/952	1.80 (1.30-2.49)		1.48 (1.05-2.09)		1.84 (1.27-2.67)		1.56 (1.06-2.31)	
Female	59/181	211/913	1.63 (1.15-2.32)		1.50 (1.03-2.17)		1.78 (1.18-2.68)		1.62 (1.06-2.49)	
**Charlson index score**				0.69		0.78		0.64		0.73
≤2	57/223	253/1380	1.57 (1.13-2.19)		1.50 (1.07-2.11)		1.69 (1.18-2.43)		1.61 (1.11-2.33)	
>2	75/151	199/485	1.43 (0.99-2.06)		1.39 (0.96-2.03)		2.31 (1.44-3.69)		2.41 (1.47-3.97)	
**Cancer diagnosis**[e]				0.75		0.64		0.77		0.61
Yes	54/104	173/462	1.79 (1.17-2.76)		1.76 (1.14-2.71)		2.16 (1.22-3.81)		2.29 (1.27-4.16)	
No	78/270	279/1403	1.65 (1.23-2.23)		1.57 (1.16-2.13)		1.76 (1.29-2.40)		1.67 (1.21-2.29)	
**Katz (ADL) index**				0.57		0.48		0.66		0.55
Independent	90/292	348/1575	1.58 (1.19-2.08)		1.37 (1.02-1.83)		1.58 (1.18-2.12)		1.35 (0.99-1.83)	
Non-independent	42/82	104/290	1.80 (1.25-3.46)		1.74 (1.03-2.96)		2.11 (0.97-4.57)		1.63 (0.71-3.78)	
**Without prior transplantation**	118/345	419/1805	1.72 (1.33-2.21)		1.47 (1.13-1.92)		1.73 (1.34-2.23)		1.47 (1.12-1.92)	

ADL, activities of daily living; IRR, incidence rate ratios.
Cases represent newly-detected colonization and/or infection with ESBL-E/CPE.
[a] IRRs were calculated using conventional logistic regression matched by age; all estimates were compared with results from conditional logistic regression.
[b] *P* values for interactions were calculated by using an interaction term.
[c] Adjusted for sex.
[d] Adjusted for sex (male, female), body mass index (continuous), Charlson Comorbidity Index score (continuous), inflammatory bowel disease (yes, no), and total duration of ICU stay (continuous) minus stratum covariate.
[e] Includes metastatic cancer.

© 2023 Willems RPJ et al. *JAMA Network Open*.

eTable 6. Additive Interaction Between Potential Microbiota-Disturbing Agents and PPI Treatment in Determining Acquisition of ESBL- or Carbapenemase-Producing Enterobacterales[a]

**Interaction with antibiotics**	**Model 1[b]**			**Model 2[c]**		
	**No PPI use**	**PPI use**		**No PPI use**	**PPI use**	
	**IRR (95% CI)**	**IRR (95% CI)**	**RERI (95% CI)[d]**	**IRR (95% CI)**	**IRR (95% CI)**	**RERI (95% CI)[d]**
No antibiotic use	1.00	1.23 (0.85-1.80)		1.00	1.06 (0.72-1.56)	
Use of antibiotics	3.01 (2.24-4.06)	4.05 (2.95-5.55)	0.80 (-0.52-2.12); *P*=0.24	2.74 (2.02-3.72)	3.42 (2.46-4.75)	0.61 (-0.56-1.78); *P*=0.31
**Interaction with immunosuppressants**	**Model 1[b]**			**Model 2[c]**		
	**No PPI use**	**PPI use**		**No PPI use**	**PPI use**	
	**IRR (95% CI)**	**IRR (95% CI)**	**RERI (95% CI)[d]**	**IRR (95% CI)**	**IRR (95% CI)**	**RERI (95% CI)[d]**
No use of immunosuppressants	1.00	1.47 (1.05-2.06)		1.00	1.24 (0.88-1.76)	
Use of immunosuppressants	1.20 (0.85-1.68)	2.17 (1.59-2.96)	0.50 (-0.32-1.32); *P*=0.23	0.95 (0.67-1.36)	1.71 (1.23-2.37)	0.51 (-0.15-1.19); *P*=0.14
**Interaction with laxatives**	**Model 1[b]**			**Model 2[c]**		
	**No PPI use**	**PPI use**		**No PPI use**	**PPI use**	
	**IRR (95% CI)**	**IRR (95% CI)**	**RERI (95% CI)[d]**	**IRR (95% CI)**	**IRR (95% CI)**	**RERI (95% CI)[d]**
No use of laxatives	1.00	1.49 (1.04-2.14)		1.00	1.35 (0.94-1.96)	
Use of laxatives	2.94 (2.16-4.01)	3.42 (2.49-4.70)	-0.01 (-1.28-1.26); *P*=0.98	2.65 (1.92-3.65)	2.81 (2.00-3.94)	-0.21 (-1.34-0.93); *P*=0.72
**Interaction with metformin**	**Model 1[b]**			**Model 2[c]**		
	**No PPI use**	**PPI use**		**No PPI use**	**PPI use**	
	**IRR (95% CI)**	**IRR (95% CI)**	**RERI (95% CI)[d]**	**IRR (95% CI)**	**IRR (95% CI)**	**RERI (95% CI)[d]**
No use of metformin	1.00	1.74 (1.35-2.26)		1.00	1.50 (1.14-1.95)	
Use of metformin	1.49 (0.68-3.26)	1.93 (1.13-3.29)	-0.30 (-1.87-1.26); *P*=0.70	1.31 (0.59-2.91)	1.53 (0.88-2.66)	-0.28 (-1.61-1.06); *P*=0.69

RERI, relative excess risk due to interaction; RERI reference value = zero; PPI, proton pump inhibitor.
[a] IRRs are calculated using conditional logistic regression matched by age.
[b] Adjusted for sex.
[c] Adjusted for sex, body mass index (continuous), Charlson Comorbidity Index score (continuous), inflammatory bowel disease (yes, no), and total duration of ICU stay (continuous).
[d] Confidence intervals for the RERI were obtained using the delta method.

© 2023 Willems RPJ et al. *JAMA Network Open*.

eTable 7. Analyses of the Acquisition of ESBL- or Carbapenemase-Producing Enterobacterales After Excluding Patients Admitted From Another Health Care Facility[a]

				Unconditional regression analysis		Conditional regression analysis	
**Exposure**	**Cases (n = 374)**	**Controls (n = 1865)**	**Total (n = 2053)**	**Sex-adjusted IRR (95% CI)**	**Adjusted IRR (95% CI)[b]**	**Sex-adjusted IRR (95% CI)**	**Adjusted IRR (95% CI)[b]**
**PPI use (0-30 d)**	115	399	514	1.78 (1.38-2.30)	1.51 (1.16-1.97)	1.87 (1.44-2.44)	1.60 (1.21-2.11)
**Acid suppression (0-30 d)[c]**							
Nonuse	218	1321	1539	1 [Reference]	1 [Reference]	1 [Reference]	1 [Reference]
Once-daily PPI	92	331	423	1.72 (1.31-2.27)	1.46 (1.10-1.95)	1.82 (1.37-2.42)	1.57 (1.17-2.11)
Twice-daily PPI	20	55	75	2.23 (1.31-3.81)	1.81 (1.05-3.2)	2.36 (1.35-4.11)	1.93 (1.09-3.43)
**PPI use (0-90 d)**	131	470	601	1.76 (1.38-2.26)	1.51 (1.16-1.96)	1.84 (1.42-2.38)	1.58 (
**Acid suppression (0-90 d)[c]**							
Nonuse	202	1250	1452	1 [Reference]	1 [Reference]	1 [Reference]	1 [Reference]
Once-daily PPI	103	388	491	1.68 (1.40-3.75)	1.44 (1.09-1.91)	1.77 (1.34-2.33)	1.52 (1.14-2.03)
Twice-daily PPI	24	66	80	2.29 (1.41-3.75)	1.89 (1.14-3.14)	2.33 (1.40-3.87)	1.96 (1.15-3.32)

Abbreviations: H2RA, histamine-2-receptor antagonist; IRR, incidence rate ratio; PPI, proton pump inhibitor.
Cases represent newly-detected colonization and/or infection with ESBL-E/CPE.
[a] IRRs were calculated using conventional logistic regression matched by age; all estimates were compared with results from conditional logistic regression.
[b] Adjusted for sex (male, female), body mass index (continuous), Charlson Comorbidity Index score (continuous), inflammatory bowel disease (yes, no), and total duration of ICU stay (continuous).
[c] Cases and controls were assigned to mutually exclusive groups based on H2RA and PPI exposure. Users of H2RAs (corresponding to 3 [0.8%] cases and 13 [0.7%] controls, and 4 [1.1%] cases and 16 [0.9%] controls, for the 0-30 days and 0-90 days window respectively) are suppressed but were included in the regression model for proper estimation of the exposure effects. Reference was always nonuse of PPI and/or H2RA.

© 2023 Willems RPJ et al. *JAMA Network Open*.

eTable 8. Analyses of the Acquisition of ESBL- or Carbapenemase-Producing Enterobacterales After Excluding Patients With Missing Values (Complete-Case Analysis)[a]

	**Unconditional regression analysis**	**Conditional regression analysis**
**Category[b]**	**Adjusted IRR ((95% CI)[c]**	**Adjusted IRR ((95% CI)[c]**
**PPI overall (0-30 d)**	1.48 (1.13-1.93)	1.54 (1.16-2.03)
Once-daily PPI	1.41 (1.06-1.87)	1.46 (1.08-1.97)
Twice-daily PPI	1.96 (1.15-3.36)	1.99 (1.13-3.52)
**PPI overall (0-90 d)**	1.48 (1.14-1.92)	1.51 (1.15-1.98)
Once-daily PPI	1.39 (1.05-1.84)	1.41 (1.05-1.90)
Twice-daily PPI	1.96 (1.19-3.23)	1.99 (1.17-3.37)

IRR, incidence rate ratio; PPI, proton pump inhibitor.
For complete cases analysis N=1658 patients were available (with N=1429 included in the conditional logistic regression analysis).
[a] IRRs were calculated using conventional logistic regression matched by age; all estimates were compared with results from conditional logistic regression.
[b] Overall and dose-response exposure to PPIs within 30 days and 90 days before the index date.
[c] Adjusted for sex, body mass index, inflammatory bowel disease, Charlson Comorbidity Index score, and ICU duration.

© 2023 Willems RPJ et al. *JAMA Network Open*.

eTable 9. Analyses of the Acquisition of ESBL- or Carbapenemase-Producing Enterobacterales With the Negative Control Exposure[a]

Negative control exposure[b]	Cases (n = 374)	Controls (n = 1865)	Adjusted IRR (95% CI)[c]
**ATC M05BA/C03AA; bisphosphonates and/or thiazide diuretics (0-30 d)**	25 (6.7)	84 (4.5)	1.35 (0.83-2.20)
Negative control exposure (ATC M05BA; bisphosphonates)	13 (3.5)	33 (1.8)	1.86 (0.95-3.62)
Negative control exposure (ATC C03AA; thiazide diuretics)	12 (3.2)	53 (2.8)	0.95 (0.48-1.89)
**ATC M05BA/C03AA; bisphosphonates and/or thiazide diuretics (0-90 d)**	32 (8.6)	111 (6.0)	1.35 (0.88-2.07)
Negative control exposure (ATC M05BA; bisphosphonates)	16 (4.3)	51 (2.7)	1.55 (0.86-2.80)
Negative control exposure (ATC C03AA; thiazide diuretics)	16 (4.3)	63 (3.4)	1.10 (0.61-1.99)

IRR, incidence rate ratio.
Cases represent newly-detected colonization and/or infection with ESBL-E/CPE.
[a] IRRs were calculated using conditional logistic regression matched by age.
[b] Overall and individual negative control exposure within 30 days and 90 days before the index date, respectively.
[c] Adjusted for sex, body mass index, inflammatory bowel disease, Charlson Comorbidity Index score, and ICU duration.

© 2023 Willems RPJ et al. *JAMA Network Open*.

eTable 10. Characteristics and Analysis of the 1:1 Matching-Pairs Population

Characteristic	Cases (n = 94)[a]	Controls (n = 94)	Sex-adjusted IRR (95% CI)	Sex, CCI, adjusted (95% CI)
Age at index date, mean (SD), y[b]	64.9 (12.8)	64.9 (12.8)	NA	NA
Age at index date, median (IQR), y[b]	65 (60-75); (range 19-87)	67 (57-73); (range 26-92)	NA	NA
<45	5 (5.3%)	5 (5.3%)	NA	NA
45-64	37 (39.4%)	37 (39.4%)	NA	NA
≥65	52 (55.3%)	52 (55.3%)	NA	NA
Female	31 (33.0%)	48 (51.1%)	NA	0.47 (0.25-0.88)
Non-Western ethnicity	11 (11.7)	5 (5.3)	2.73 (0.82-9.12)	2.75 (0.82-9.23)
Hospital department				
Medical	74 (78.7)	82 (87.2)	-	-
Surgical	13 (13.8)	9 (9.6)	-	-
Emergency department	7 (7.5)	3 (3.2)	-	-
Admitted from a healthcare facility	10 (10.6%)	3 (3.2%)	3.90 (1.02-14.9)	3.94 (1.02-15.2)
International travel (<6 months)[c]				
Yes	11 (11.7)	19 (19.2)	1.04 (0.41-2.66)[f]	1.05 (0.41-2.69)[f]
No	83 (88.3)	75 (79.8)	1 [Reference]	1 [Reference]
Alcohol use[c]				
Yes	24 (25.5)	20 (42.5)	0.78 (0.38-1.61)[f]	0.79 (0.38-1.63)[f]
No	70 (74.5)	74 (78.7)	1 [Reference]	1 [Reference]
Current smoking[c]				
Yes	4 (4.3)	8 (8.5)	0.74 (1.67-3.26)[f]	0.74 (0.17-3.30)[f]
No	90 (95.7)	86 (91.5)	1 [Reference]	1 [Reference]
BMI (kg/m2), mean (SD)[c]	24.8 (4.5)	25.4 (4.8)		
BMI (kg/m2), mean (SD)	26.5 (5.4)	26.0 (5.5)	1.03 (0.97-1.10)[e]	1.03 (0.97-1.10)[e]
<25	38 (45.2)	33 (45.2)	1 [Reference]	1 [Reference]
25-30	31 (36.9)	23 (31.5)	0.93 (0.48-1.81)[e]	0.93 (0.48-1.82)[e]
≥30	15 (17.9)	17 (23.3)	0.97 (0.44-2.11)[e]	0.97 (0.44-2.15)[e]
CCI score, median (IQR)	2 (0-4)	2 (0-3)	1.00 (0.91-1.10)	1.00 (0.91-1.10)
0	30 (31.9)	38 (40.4)	1 [Reference]	1 [Reference]
1-2	27 (28.7)	22 (23.4)	-	1.59 (0.76-3.33)
>2	37 (39.4)	34 (36.2)	-	1.10 (0.54-2.25)
Inflammatory bowel disease	7 (7.5)	6 (6.4)	1.25 (0.41-3.83)	1.25 (0.39-3.98)
Katz (ADL) index, mean (SD)	5.5 (1.2)	5.5 (1.2)	0.98 (0.77-1.26)	0.99 (0.76-1.28)
Prior hospitalization (<6 months)	41 (43.6)	44 (46.8)	0.91 (0.50-1.68)	0.89 (0.46-1.73)
Prior ICU admission	18 (19.1)	5 (5.3)	4.86 (1.58-15.0)	4.86 (1.58-15.0)
Days in the ICU, median (IQR)	4 (2-12); (range 1-32)	5 (2-7); (range 2-9)	1.22 (0.98-1.53)	1.2 (0.98-1.53)
Prior hospitalization (<6 months)[c]				
Yes	39 (41.5)	54 (57.5)	0.93 (0.39-2.24)[f]	0.93 (0.38-2.23)[f]
No	55 (58.5)	40 (42.5)	1 [Reference]	1 [Reference]
Surgery (<90 days)	19 (20.2)	22 (23.4)	0.65 (0.29-1.42)	0.62 (0.28-1.41)

© 2023 Willems RPJ et al. *JAMA Network Open*.

Solid organ or stem cell transplantation (<90 days)	4 (4.3)	9 (9.6)	0.33 (0.10-1.13)	0.32 (0.09-1.11)
Medication[g]				
Antirheumatic drugs	3 (3.2)	4 (4.3)	0.51 (0.10-2.50)	0.49 (0.10-2.49)
Metformin use	5 (5.3)	5 (5.3)	0.67 (0.16-2.86)	0.64 (0.14-2.90)
Laxative use	48 (51.1)	27 (28.7)	3.4 (1.62-7.02)	3.43 (1.64-7.2)
Immunomodulating drug	39 (41.5)	27 (28.7)	1.48 (0.81-02.71)	1.49 (0.81-2.75)
Antibiotic (≥1)	45 (47.9)	37 (39.4)	1.43 (0.77-2.66)	1.45 (0.77-2.76)
PPI[d]	41 (43.6)	29 (30.9)	2.19 (1.02-4.73)	2.28 (1.04-5.00)
Negative control (<30 days)	6 (6.4)	6 (6.4)	1.00 (0.27-3.63)	1.00 (0.28-3.64)
Thiazide diuretics	3 (3.2)	3 (3.2)	1.14 (0.22-5.96)	1.14 (0.22-5.96)
Bisphosphonates	3 (3.2)	3 (3.2)	0.88 (0.17-4.60)	0.88 (0.17-4.61)

ADL, activities of daily living; BMI, body mass index ([calculated as weight in kilograms divided by height in meters squared]); CCI, Charlson Comorbidity Index; H2RA, histamine-2 receptor antagonist; ICU, intensive care unit; IRR, incidence rate ratio; IQR, interquartile range; LOS, length of stay; PPI, proton pump inhibitor; SD, standard deviation.
Unless otherwise indicated, data are expressed as number (percentage) of participants.
[a] Of the 94 cases, 7 (7.5%) were positive within 48 hours of admission and 87 (92.6%) were positive after 48 hours of hospitalization.
[b] Matching variable.
[c] As measured by questionnaire.
[d] Within 30 days before the index. There were insufficient users of a twice-daily PPI regimen to allow for a reasonable dose-response analysis.
[e] For this analysis, BMI values were missing for 31 (16.5%; 10 [10.6%] cases and 21 [22.3%] controls), these were imputed using the self-reported BMI values (18 [9.5%]) and the population median 25.7 (IQR 22.6-28.8) otherwise (13 [6.9%]).
[f] Estimated using a separate category for missing values.
[g] Only 1 patient used a H2RA (control patient).

© 2023 Willems RPJ et al. *JAMA Network Open*.

eTable 11. Analyses of the Acquisition of ESBL- or Carbapenemase-Producing Enterobacterales in the 1:1 Matching-Pairs Case-Control Study

**Analysis**[b]	**Adjusted IRR (95% CI)**[a]
	**30-day time window**
**Model 1**	2.96 (1.14-7.74)
**Model 2**	3.08 (1.16-8.13)
**Model 3**	2.88 (1.09-7.60)
**Model 4**	2.87 (1.09-7.48)
**Model 5**	3.10 (1.15-8.37)
**Model 6**	2.83 (1.01-7.91)

IRR, incidence rate ratio.
[a] Estimated using conditional logistic regression matched by age.
Model 1: Adjusted for age, sex, BMI, Charlson Comorbidity Index score, inflammatory bowel disease, ICU duration, and transplantation.
Model 2: Adjusted for age, sex, BMI, Charlson Comorbidity Index score, inflammatory bowel disease, ICU duration, cephalosporin use and transplantation.
Model 3: Adjusted for age, sex, BMI, Charlson Comorbidity Index score, inflammatory bowel disease, ICU duration, prior hospitalization, surgery, and transplantation.
Model 4: Adjusted for model 1 plus travel.
Model 5: Adjusted for model 1 plus ethnicity.
Model 6: Adjusted for model 1 plus alcohol intake and smoking.

© 2023 Willems RPJ et al. *JAMA Network Open*.

eTable 12. Analysis of Misclassification

To assess the effect of misclassification of controls on the primary estimate of our analysis, we conducted a quantitative bias analysis. Possible misclassification could have occurred due to controls not having faecal swabs taken; therefore, some controls may not be recognized as cases. It would be expected that approximately 5 to 10% of controls would carry detectable ESBL-E/CPE in the gut (if fecal specimens had been taken).

479 controls were correctly classified, because faecal cultures were available.
5 to 10% of 1386 controls may have been misclassified, because they had no fecal culture. We added the calculation for 15% as extra margin.

The exposure frequency of the controls was reweighted, assuming that 5, 10 or 15% of controls without fecal cultures might have been cases (as cases had a higher frequency of exposure, the reweighted exposure frequency of the controls became lower than measured). This resulted in the following IRRs, adjusted for misclassification.

	***k* = 5%**	***k* = 10%**	***k* = 15%**
	**IRR_1**	**IRR_2**	**IRR_3**
**$IRR_{bias\text{-}adjusted}$**	1.75	1.79	1.81

k, proportion of misclassification

This analysis showed that the bias-adjusted IRRs were marginally larger than the original IRR when taking misclassified controls into account.

The calculations were based on Chyou, Lash et al, and Groenwold et al.[19-20]

© 2023 Willems RPJ et al. *JAMA Network Open*.

eFigure 1. Visualization of the Study Design and Time Frame[a]

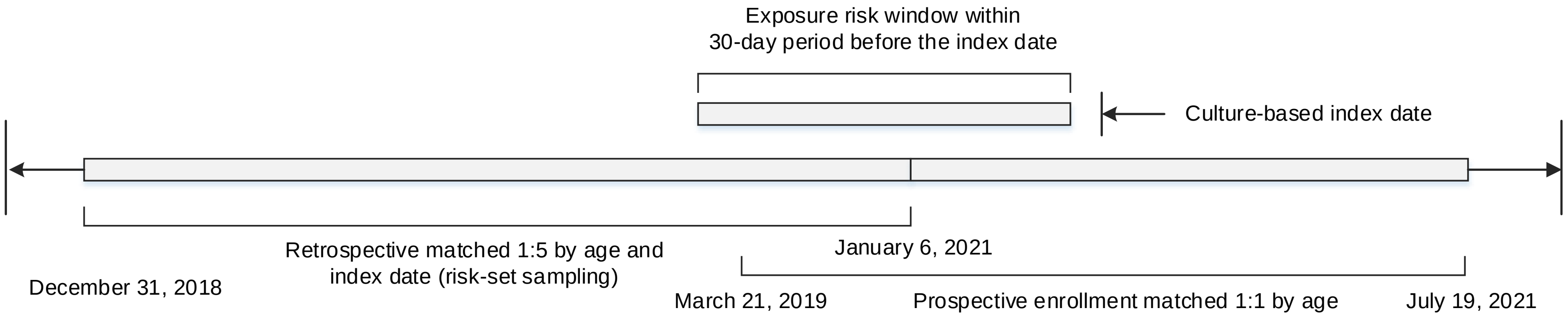

[a] Cases and controls (≥18 years) were matched on age, because older patients with multi-morbidity are more likely to use proton pump inhibitors (PPIs); and, additionally, older patients are more likely to be exposed to health care settings and to antibiotics. For each case, 5 controls were identified and matched on the date the case tested positive for newly-detected extended-spectrum β-lactamase or carbapenemase-producing Enterobacterales (no known history of ESBL-E/CPE) isolates in clinical cultures using risk-rest sampling. Eligible cases had a first positive ESBL-E/CPE culture during 2018-2021, and no ESBL-E/CPE positive isolates during 2015 -2017, ensuring a ≥3-year history.

© 2023 Willems RPJ et al. *JAMA Network Open*.

eFigure 2. Directed Acyclic Graph[a]

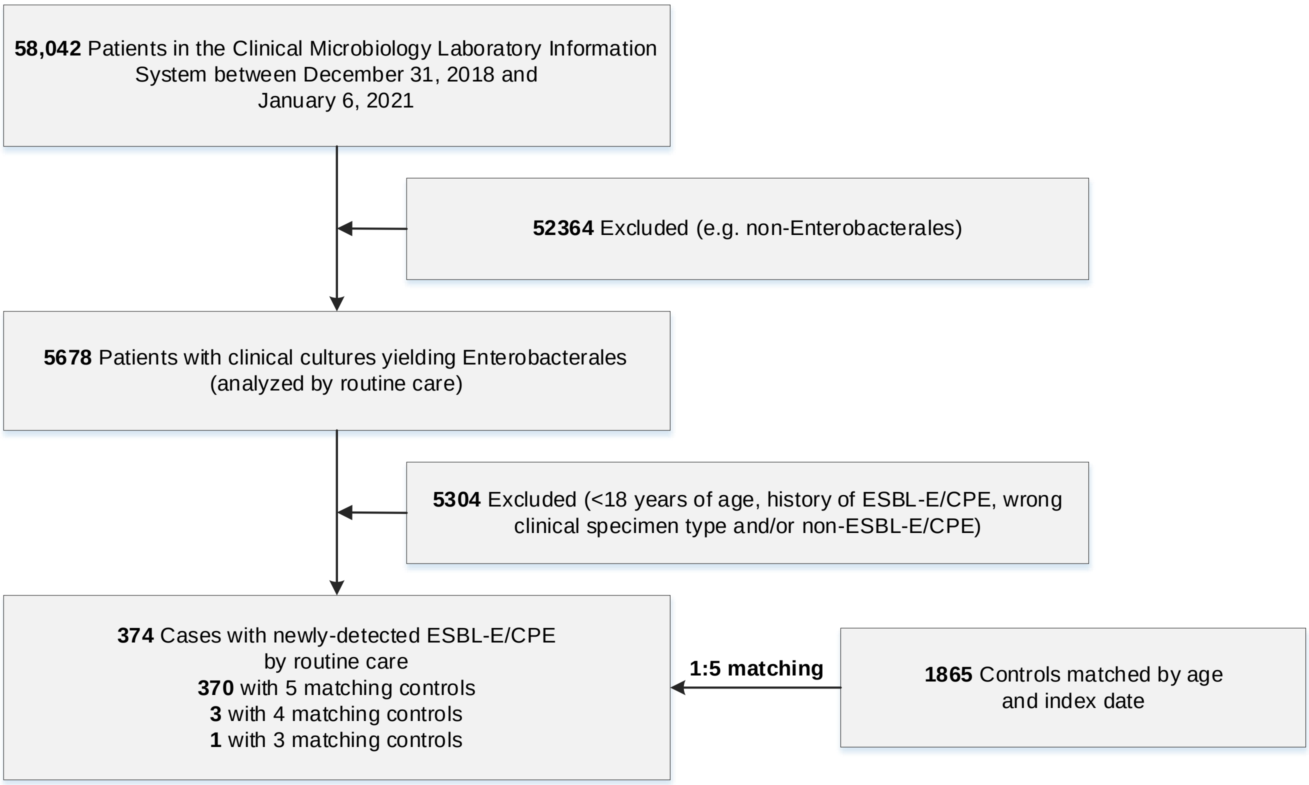

ESBL-E/CPE, extended-spectrum β-lactamase or carbapenemase-producing Enterobacterales.
[a] Cases and controls were matched on age (±5 years) and the date the case tested positive (±15 days) via risk-set sampling without replacement. The case date of ESBL-E/CPE confirmation was assigned the index date. Eligible controls were those with a culture negative for ESBL-E/CPE analyzed by routine care – controls were selected from the eligible cohort. Three cases had 4 matched controls, and 1 case had 3 matched controls.

© 2023 Willems RPJ et al. *JAMA Network Open*.

eFigure 3. Flow Diagram of the Nested 1:5 Matched Design

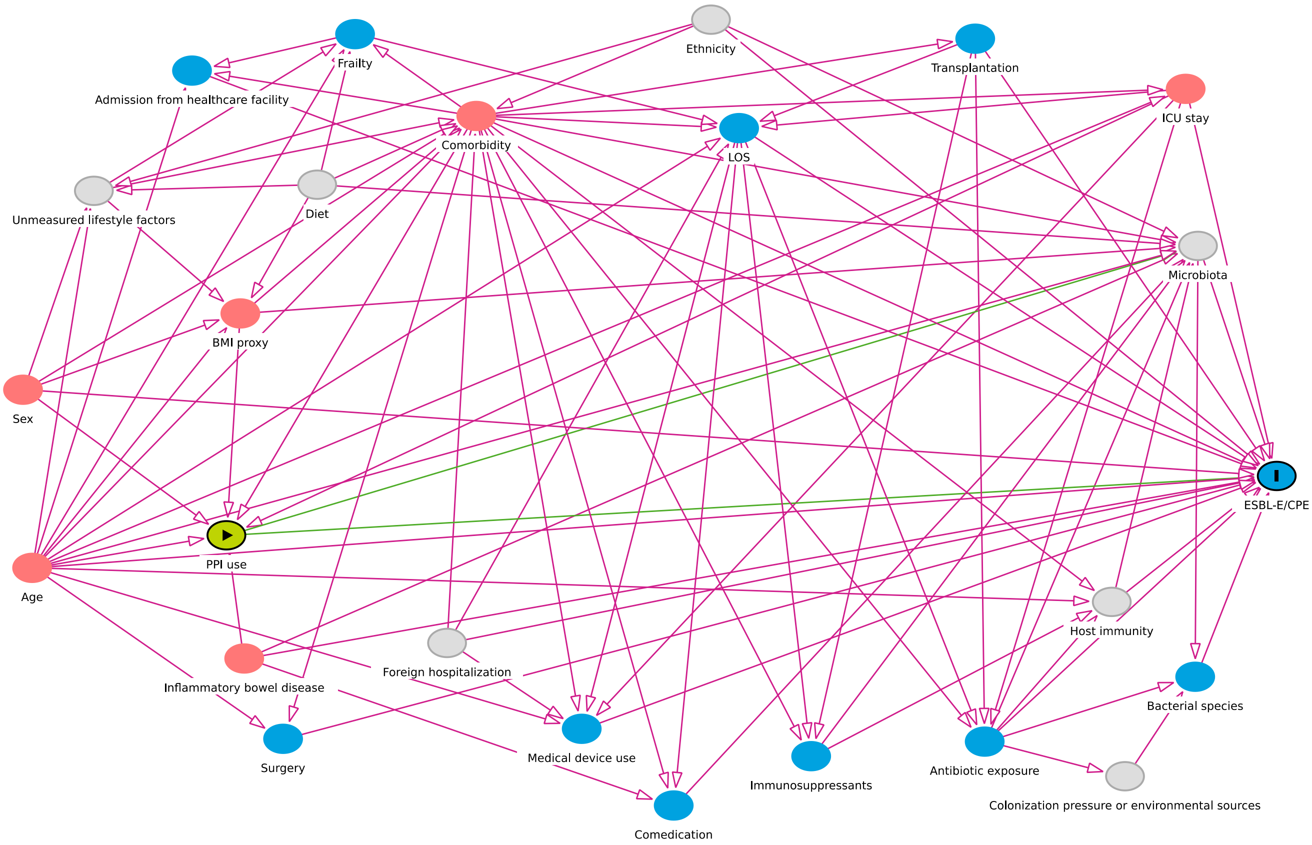

LOS, hospital length of stay, ICU, intensive care unit, BMI, body mass index, ESBL-E/CPE, extended-spectrum β-lactamase or carbapenemase-producing Enterobacterales.
Gray circles indicate unobserved (latent) variables, blue circles indicate potential mediators (causal determinants of the outcome) and red circles indicate confounders. Pink lines depict biasing paths.
BMI and the CCI score were used as proxy confounders of unobserved lifestyle factors under the proximal framework.
Inflammatory bowel disease was considered a marker of frequent hospitalization, exposure to antibiotic or immunosuppressive treatment, and gastrointestinal dysbiosis, which may render these patients prone to colonization with ESBL-E/CPE. Given overall antibiotic exposure is in the pathway and not suggested as a confounding variable for the relationship between PPI and ESBL-E/CPE acquisition, adjustment could potentially bias the results towards the null. Since we did not observe a clear interaction, we adjusted for cephalosporin use to assess the stability of our results. The importance of other factors was assessed by determining whether their addition modified the OR by ≥10% (e.g. transplantation, the use of immunosuppressants, bacterial species or type of culture).

© 2023 Willems RPJ et al. *JAMA Network Open*.

eFigure 4. Flow Diagram of the 1:1 Matching-Pairs Study (Prospectively Enrolled)[a]

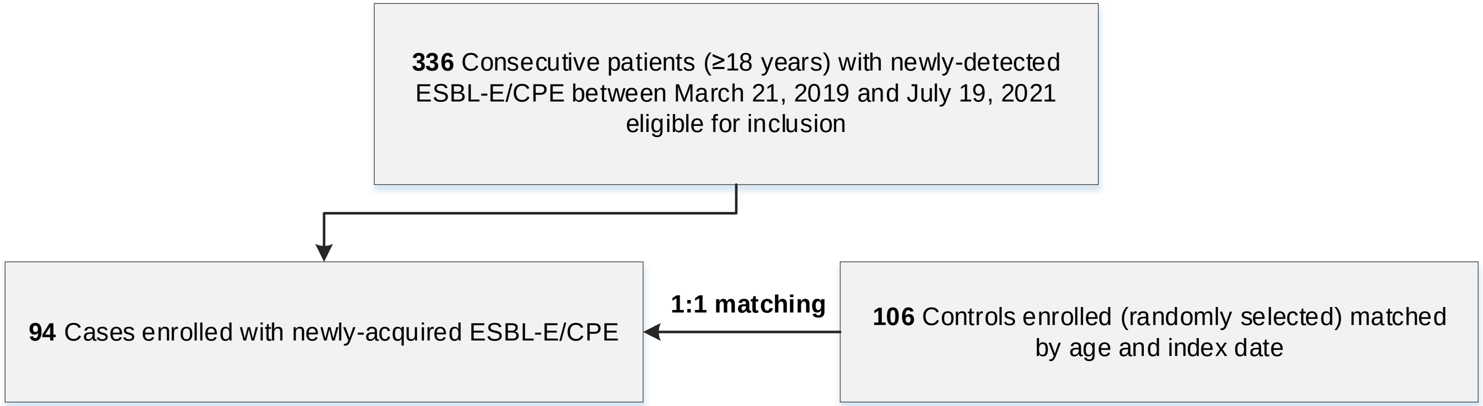

ESBL-E/CPE, extended-spectrum β-lactamase or carbapenemase-producing Enterobacterales.
[a] To evaluate unmeasured confounders not recorded in the medical records, we conducted a study of consecutive cases prospectively enrolled. The analysis was performed using data from 94 consecutive cases matched to 94 randomly selected controls (the excluded unmatched controls had a median [IQR] age of 64.5 [40-71]; 38.9% were female). The in-, exclusion and matching criteria were the similar to the retrospective study. Recruitment to the preplanned sample size was hampered as a result of restrictions imposed during the national SARS-CoV-2 lockdown. Population not enrolled: mean age of 60.4 (SD, 17.1); 44% were female.

© 2023 Willems RPJ et al. *JAMA Network Open*.

eReferences

1. Magiorakos AP, Srinivasan A, Carey RB, et al. Multidrug-resistant, extensively drug-resistant and pandrug-resistant bacteria: an international expert proposal for interim standard definitions for acquired resistance. *Clin Microbiol Infect.* 2012;18(3):268-81. doi:10.1111/j.1469-0691.2011.03570.x
2. European Committee on Antimicrobial Susceptibility Testing. EUCAST Guidelines for Detection of Resistance Mechanisms and Specific Resistances of Clinical and/or Epidemiological Importance, Version 2.0. Basel, Switzerland: EUCAST; 2017
3. van der Zwaluw K, de Haan A, Pluister GN, Bootsma HJ, de Neeling AJ, Schouls LM. The carbapenem inactivation method (CIM), a simple and low-cost alternative for the Carba NP test to assess phenotypic carbapenemase activity in gram-negative rods. *PLoS One.* 2015;10(3):e0123690. doi:10.1371/journal.pone.0123690
4. Lu H, Cole SR, Platt RW, Schisterman EF. Revisiting Overadjustment Bias. *Epidemiology.* 2021;32(5):e22-e23. doi:10.1097/ede.0000000000001377
5. Tchetgen EJT, Ying A, Cui Y, Shi X, Miao W. An Introduction to Proximal Causal Learning. *medRxiv.* 2020:2020.09.21.20198762. doi:10.1101/2020.09.21.20198762
6. Bradford PA. Extended-spectrum beta-lactamases in the 21st century: characterization, epidemiology, and detection of this important resistance threat. *Clin Microbiol Rev.* 2001;14(4):933-51, table of contents. doi:10.1128/cmr.14.4.933-951.2001
7. Paterson DL, Bonomo RA. Extended-spectrum beta-lactamases: a clinical update. *Clin Microbiol Rev.* 2005;18(4):657-86. doi:10.1128/cmr.18.4.657-686.2005
8. Pitout JD, Laupland KB. Extended-spectrum beta-lactamase-producing Enterobacteriaceae: an emerging public-health concern. *Lancet Infect Dis.* 2008;8(3):159-66. doi:10.1016/s1473-3099(08)70041-0
9. Schwaber MJ, Klarfeld-Lidji S, Navon-Venezia S, Schwartz D, Leavitt A, Carmeli Y. Predictors of carbapenem-resistant Klebsiella pneumoniae acquisition among hospitalized adults and effect of acquisition on mortality. *Antimicrob Agents Chemother.* 2008;52(3):1028-33. doi:10.1128/aac.01020-07
10. VanderWeele TJ, Knol MJ. A tutorial on interaction. *Epidemiol Methods.* 2014;3(1):33-72. doi:10.1515/em-2013-0005
11. Rothman KJ, Greenland S. Planning Study Size Based on Precision Rather Than Power. *Epidemiology.* 2018;29(5):599-603. doi:10.1097/ede.0000000000000876
12. Huizinga P, van den Bergh MK, van Rijen M, Willemsen I, van 't Veer N, Kluytmans J. Proton Pump Inhibitor Use Is Associated With Extended-Spectrum β-Lactamase-Producing Enterobacteriaceae Rectal Carriage at Hospital Admission: A Cross-Sectional Study. *Clin Infect Dis.* 2017;64(3):361-363. doi:10.1093/cid/ciw743
13. Castor EDC. Castor Electronic Data Capture. 2019. [online] Available at: https://castoredc.com. Castor Electronic Data Capture. Ciwit BV. Amsterdam, the Netherlands; 2018
14. White IR, Royston P, Wood AM. Multiple imputation using chained equations: Issues and guidance for practice. *Stat Med.* 2011;30(4):377-99. doi:10.1002/sim.4067
15. Sterne JA, White IR, Carlin JB, et al. Multiple imputation for missing data in epidemiological and clinical research: potential and pitfalls. *BMJ.* 2009;338:b2393. doi:10.1136/bmj.b2393
16. Rubin, DB. Multiple Imputation for Nonresponse in Surveys. New York: John Wiley & Sons, 1987
17. Wacholder S. The case-control study as data missing by design: estimating risk differences. *Epidemiology.* 1996;7(2):144-50. doi:10.1097/00001648-199603000-00007
18. Chyou PH. Patterns of bias due to differential misclassification by case-control status in a case-control study. *Eur J Epidemiol.* 2007;22(1):7-17. doi:10.1007/s10654-006-9078-x.
19. Lash TL, Fox MP, MacLehose RF, Maldonado G, McCandless LC, Greenland S. Good practices for quantitative bias analysis. *Int J Epidemiol.* 2014;43(6):1969-85. doi:10.1093/ije/dyu149
20. Groenwold RHH, Dekkers OM. Measurement error in clinical research, yes it matters. *Eur J Endocrinol.* 2020;183(3):E3-E5. doi:10.1530/EJE-20-0550.

© 2023 Willems RPJ et al. *JAMA Network Open*.
