# Supplementary material for: Association of Proton Pump Inhibitor Use With Risk of Acquiring Drug-Resistant Enterobacterales
Source: JAMA Netw Open. 2023 Feb 23;6(2):e230470. doi: 10.1001/jamanetworkopen.2023.0470 (PMC9951039; doi:10.1001/jamanetworkopen.2023.0470)
Supplement: Supplement 2. — Data Sharing Statement [file jamanetwopen-e230470-s002.pdf]

Data Sharing Statement

Willems. Association of Proton Pump Inhibitor Use With Risk of Acquiring Drug-Resistant Enterobacterales. *JAMA Netw Open*. Published February 23, 2023.
doi:10.1001/jamanetworkopen.2023.0470

Data
Data available: No
